# Forecasting electricity consumption of India through nighttime satellite imagery

**DOI:** 10.1371/journal.pone.0327031

**Published:** 2025-09-25

**Authors:** Darshini R., Akshay Kumar, Maria Anu Vensuslaus, Rishikeshan C. A., Joshua Thomas John Victor

**Affiliations:** 1 Student, School of Computer Science and Engineering, Vellore Institute of Technology, Chennai, India; 2 Faculty, School of Computer Science and Engineering, Vellore Institute of Technology, Chennai, India; 3 Faculty, Engineering Computing & Built Environment, UOW Malaysia KDU University College, Shah Alam, Malaysia; Khalifa University of Science and Technology, UNITED ARAB EMIRATES

## Abstract

Amidst a growing need for effective energy management, government policies increasingly rely on accurate electricity consumption forecasts to make informed decisions on renewable energy adoption. This study investigates the predictive capabilities of night light satellite imagery in forecasting electricity usage in India, aligning with Sustainable Development Goals 7 and 10. Utilizing data from the VIIRS satellite and NASA’s Black Marble product, the research employs various LSTM models to analyse electricity consumption trends. Additionally, state-wise analyses have been conducted by applying k-means clustering to capture spatial consumption variations. By demonstrating the strong correlation between night lights and electricity consumption, the study emphasizes the utility of satellite imagery for actionable insights into energy dynamics. The results emphasize the viability of night light data as a dependable indicator of electricity demand, with MAPE values below 10% and RMSE values below 20 MU. It also highlights the transformative impact of remote sensing technologies in advancing sustainable development agendas and highlights the pivotal role of night light imagery in energy forecasting initiatives.

## 1. Introduction

Electricity stands as an essential pillar of modern civilization, facilitating every aspect of daily life, from household amenities to industrial processes and communication networks. In India, with a huge population and diverse industrial landscape, the electricity sector plays a pivotal role in supporting economic growth and improving living standards. India’s electricity sector is a vital component of its infrastructure, playing a crucial role in powering its rapidly growing economy and supporting the daily lives of its citizens.

With a population of over 1.3 billion people with projections of up to 1.6 billion by 2040 and a diverse range of industries and regions, India faces unique challenges and opportunities in meeting its electricity needs. India naturally emerges as a significant consumer of electric power due to the direct correlation between electricity consumption and population size. India also emerges as the 2^nd^ largest coal consumer and the 4^th^ largest emitter of CO_2_. Despite progress, challenges like electricity shortages and distribution losses persist, urging the need for modernization and sustainable energy solutions.

The Sustainable Development Goals (SDGs) highlight the importance of electricity access and sustainability, underscoring the need for clean and affordable energy. India’s significant electricity consumption, largely fuelled by coal, makes it crucial to manage energy demand efficiently [1]. Therefore, for a vast country like India, there is an increasing need to precisely forecast future energy demands which would facilitate efficient allocation and utilization of resources. Additionally, there is a requirement to provide clean and affordable energy to all, thereby reducing greenhouse gas emissions.

This human activity of electricity consumption manifests visibly beyond Earth, as satellite imagery captures nighttime light emissions, commonly referred to as night light satellite images These images, obtained through remote sensing techniques, serve a pivotal role in assessing the impact of electricity consumption across diverse regions. Night light satellite imagery emerges as a valuable tool for assessing electricity consumption and socio-economic indicators [2,3]. While past research has primarily focused on China, there is a gap in understanding India’s electricity dynamics at both national and state levels. While primarily utilized for gauging electric consumption, this imagery also provides insights into various socio-economic indicators such as GDP, poverty levels, and population distribution.

The initial program, conducted by the US Air Force Defence Meteorological Satellite Program (DMSP) Operational Linescan System (OLS), lacked a digital archive for the first 20 years of operation. However, digital archives were established in 1992, enabling the scientific community to actively contribute to research in this field. The DMSP-OLS stopped their operations in 2013, resulting in temporal coverage from 1992 to 2013. Subsequently, the Visible Infrared Imaging Radiometer Suite (VIIRS) emerged as the primary data source for night light satellite imagery, commencing in 2012. VIIRS offers superior resolution compared to DMSP and has extensive temporal coverage from 2012 to the present.

One of the foremost research projects in this field was done by Chand et al. [4] where they utilized DMSP-OLS satellite imagery to generate stable light images, enabling the assessment of spatial changes in night-time lights and their correlation with electricity consumption in India from 1992 to 2003. Their study demonstrated the potential of night light data to analyse regional shifts in electric power consumption, offering insights for improving predictions of electricity consumption patterns. Jasinski’s study [5] aimed to forecast annual electricity usage at the NUTS-2 level in Polish regions using VIIRS DNB nighttime data, highlighting its advantages over DMSP-OLS imagery. By integrating spatial development indicators and employing both linear regression models and ANNs, Jasinski demonstrated the superiority of neural networks in predicting electricity consumption, emphasizing their ability to capture the complex, non-linear relationships between variables.

Sahoo et al. [6] compared DMSP-OLS and VIIRS methods for estimating EPC in Uttar Pradesh, India. They focused on calibrating DMSP-OLS annual composites for 2013 using the Ridgeline Sampling Regression (RSR) method and primarily utilized Ordinary Least Squares method to estimate Electric Power Consumption (EPC) coefficients. In a study conducted by Lin et al. [7] they addressed data gaps and noise in the (Night Time Light) NTL dataset, applied spatial filtering techniques, and calculated cumulative NTL values for each provincial region, ultimately utilizing regression models to establish a robust correlation between NTL data and EPC for accurate analysis of energy usage patterns.

Falchetta et al. [8] addressed calibration inconsistencies and sensor noise in NTL data by modifying cells with very low or negative radiance values to zero and also correcting disproportionately high radiance values. They calculated median annual radiance values for each pixel and integrated these statistics with electricity consumption and per-capita Gross National Income. Log-log regressions incorporating fixed-effects for country and year were conducted to evaluate the relationship between NTL and electricity consumption, aiming to predict interannual variations in per-capita electricity consumption figures across income categories and macro-regions.

Li et al. [9] estimated electricity consumption in China by classifying cities based on statistical employment data across 19 sectors using cluster analysis. They employed a k-means algorithm for city classification and utilized linear regression with Weighted Least Squares to model the relationship between EPC and Total Night Lights (TNL), with weights assigned to address heteroscedasticity. Shi et al. [10] utilized DMSP-OLS data to estimate EPC and establish correlations with regional EPC using linear regression models. They further characterized spatiotemporal variations in EPC, assessed directional bias, evaluated EPC distribution among Belt and Road countries, and conducted correlation analysis with GDP and population, providing comprehensive insights into the dynamics and correlations surrounding EPC in these regions. The methodologies discussed predominantly relied on linear regressions or conventional statistical methods, with only one study utilizing neural networks for analysis. While [10] utilized spatial disparities to estimate NTL, the estimation of EPC in most studies continued to rely on linear regression.

In their study [11, 12], Beyer et al. evaluated the effectiveness of nighttime lights as indicators of economic activity in India. Their analysis revealed that increase in electricity consumption corresponded to significant growth in Gross Value Added, with electricity consumption serving as a robust indicator at both national and state levels while nighttime light intensity proved useful for tracking economic activity at the district and city levels. Dasgupta’s study [13] differed by incorporating both NTL and EPC as predictors to gauge economic activity in India during COVID. Their approach involved three steps: filtering nightlight radiance values to address artifacts, employing panel regression to estimate the elasticity of NTL and EPC concerning GDP, and utilizing machine learning techniques to estimate the economic impact of COVID. Both of these studies enhanced their analysis by incorporating additional socio-economic indicators such as GDP and population along with NTL.

Rahman et al.‘s study [12] employed various statistical approaches, including linear regression, multiple linear regression, exponential smoothing techniques, and curve estimation techniques, to forecast EPC in India. By considering factors like GDP, population, and GDP per capita, alongside sector-specific data, the study aimed to provide reliable forecasts for informed policy-making and planning. Khan et al. [14] developed a novel approach combining CNN with LSTM-AE models to enhance electricity consumption prediction efficiency in both residential and commercial structures. Their framework involved CNN for spatial feature extraction, LSTM for capturing long-term dependencies, and LSTM-AE for unsupervised learning and feature representation, offering a reliable solution for accurate electricity consumption prediction and aiding in effective energy management.

Yan et al. [15] aimed to enhance the precision and resilience of forecasting electricity usage in individual households using a hybrid deep learning approach comprising LSTM and CNN. Their framework preprocesses input data with temporal CNN for efficient feature extraction before feeding it into LSTM units for forecasting, introducing an innovative strategy for power consumption forecasting that predicts multiple future data points concurrently, thus improving upon conventional single-step forecasting methods. Klyuev et al. [16] conducted a review on methods of forecasting electricity consumption, highlighting the methodologies employed across various forecasting horizons. Their findings underscore the significance of employing a blend of classical and intelligent approaches tailored to specific forecasting horizons to ensure accurate and informed decision-making in the dynamic energy sector.

Somu et al. [17] provided an overview of energy consumption forecasting, emphasizing the utilization of k-means clustering, LSTM, and CNNs. Their approach involves utilizing k-means clustering for partitioning cluster analysis, and integrating CNN and LSTM networks in a CNN-LSTM model architecture to forecast building energy consumption by capturing spatial correlation and temporal information from irregular consumption patterns. Bilgili et al. [18] investigated estimating electricity consumption in Turkey, focusing on employing LSTM models for accurate forecasts.

Most of the studies show a predominant reliance on statistical modelling techniques, particularly linear regression, for estimating electric power consumption using night light data. While some studies incorporate additional variables such as GDP and population, few explore forecasting future electricity demands. Moreover, existing forecasting studies often focus solely on EPC without considering other factors, and linear models may not adequately capture the non-linear relationship between night light data and electricity consumption.

To address these gaps, this study concentrates on forecasting electric power consumption in India by leveraging nighttime satellite images. State-wise analysis is conducted to capture spatial variations, with clustering algorithms employed to enhance estimation accuracy. By focusing on the Indian context and incorporating spatial variation, this research aims to provide insights into electric power consumption dynamics at a granular level, contributing to a more comprehensive understanding of energy usage patterns in India. This study also aims to fill this void by employing deep learning techniques like LSTM to enhance forecasting accuracy [19]. By identifying optimal models capable of capturing complex data patterns, this study seeks to aid in making accurate forecasts that would help in making informed policy formulations and promote sustainable energy management in India.

The key contributions of the study are as follows:

1. Utilizing night-time lights captured through satellite imagery as an additional predictor variable to enhance the accuracy of electric power consumption forecasting in India, an area with limited prior research.
2. Implementing deep learning neural networks like LSTM for more accurate forecasting, surpassing the limitations of linear regressions and traditional statistical models by providing end-to-end forecasting solutions.
3. Integrating spatial variation through state-wise analysis to improve the precision and reliability of forecasts and predictions.

The following sections are structured as follows: Section 2 addresses data collection and pre-processing methods, Section 3 discusses the methodology and proposed system, Section 4 presents the results and discussion, followed by Section 5, which discusses the conclusion and outlines future work.

## 2. Data collection and preprocessing

### 2.1. Satellite data processing

#### 2.1.1. Background.

Night light satellite images have been widely employed in various studies to quantify socio-economic parameters. These NTL images are particularly useful for estimating electricity consumption in specific geographic regions. Over time, multiple satellite missions have been conducted to capture NTL imagery but the most significant missions with freely available data include those from the DMSP-OLS and VIIRS programs.

Most recent studies utilize VIIRS data due to its superior image quality compared to DMSP-OLS, attributed to enhanced sensor resolution and calibration enhancements [20]. VIIRS effectively addresses issues such as blooming and saturation effects, while also providing a spatial resolution of 500 meters. However, it is noteworthy that VIIRS’ overpass time is at 01:30 UTC nightly, which may result in some significant loss of peak night lights.

To harness the potential of the VIIRS Day/Night Band (DNB) dataset, NASA has introduced a set of products known as the Black Marble product suite (VNP46/VJI46). This suite comprises 4 distinct product sets generated by the Black Marble algorithm. This study uses the VNP46A2 and VNP46A4 product suites for the daily and yearly night light changes respectively.

The VNP46A2 product applies the Black Marble Algorithm to improve the quality of generated nighttime light images by correcting for atmospheric, terrain, lunar BRDF, thermal, and straylight influences. Through this process, the nighttime radiance is enhanced, leading to better detection of nighttime lights over quicker time spans and a decrease in the noise [20].

The VNP46A2 product offers 7 scientific datasets [20,21] with a resolution of 15 arc seconds, covering the period from January 2012 to the present, providing valuable data for analysing long-term trends [22] in nighttime light series. Its daily moonlight and atmospheric corrected data, along with innovative noise reduction techniques like the “Turn off the moon” approach [23], contribute to high-quality data suitable for this study. This data coupled with corrective measures, significantly contribute to reducing background noise and distortion, resulting in high-quality data [24]. The VNP46A4 dataset is derived by aggregating data from the daily VNP46A2 dataset. It is available at a resolution of 15 arc seconds and encompass a temporal range from January 2012 to the present. Each composite product comprises 28 layers, providing information on various aspects of the dataset.

#### 2.1.2. Night light image extraction.

The NTL data processing of the VNP46A2 product has been performed in Google Earth Engine (GEE) interface, used for remote sensing applications. The daily raster files are loaded for days from January 19^th^, 2012 till 31^st^ December, 2023. For this study the two layers: ‘Gap Filled DNB BRDF Corrected NTL’ and the ‘Mandatory Quality Flag’ were extracted. Only those pixels with quality flag values as 0 or 1 (Table 1 in S1 File), representing good quality pixels were taken for further processing. As a part of the preprocessing steps, pixels with radiance values less than 0.5 nWcm^-2^sr^-1^ and extremely high radiance values were removed to reduce the background noise [25].

**Fig 1:**
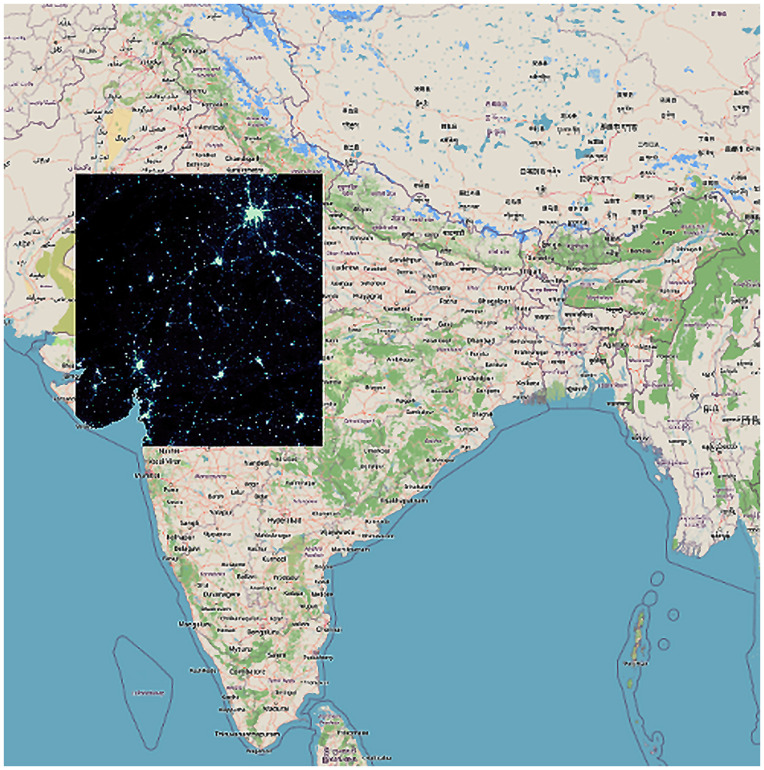
Single tile. Geographic Tiles Covering India.

**Fig 2:**
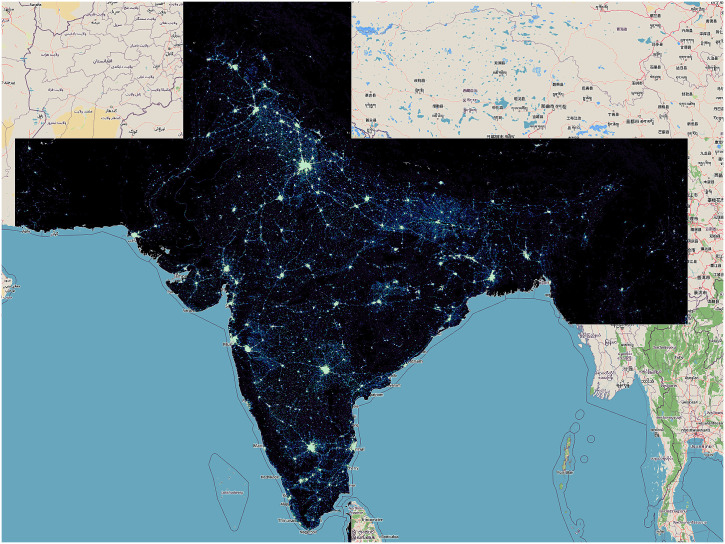
8 tiles. Geographic Tiles Covering India.

VNP46A4 was not accessible on GEE like VNP46A2, hence alternative measures were taken to obtain data for the geographic region of India. Individual tiles covering India’s geographic extent (h24v6, h25v5, h25v6, h25v7, h25v8, h26v6, h26v7) were downloaded from NASA’s LAADS – DAAC website [26,27] (Figs 1 and 2). Each of these tiles was downloaded monthly from January 2012 to December 2023 and on a yearly basis for the years 2012–2023.

Each tile is stored in HDF5 (.h5) format, which has files arranged into various folders. Within each HDF5 file, there is a geocoded dataset called GeoTIFF, represented as a matrix with dimensions of 2400 rows by 2400 columns. VNP46A4 has 28 layers stored in GeoTIFF format and the layers ‘All Angle Composite Snow Free’ and ‘All Angle Composite Snow Free Quality’ were utilized in this study and were extracted using R script. The geographic extent of each tile was calculated using its horizontal and vertical tile number.

To facilitate further analysis and extract meaningful information, the QGIS application was employed. Initially, all tiles covering the geographic area of India were mosaicked and merged within the application. Subsequently, the merged raster layer underwent raster clipping, where the image was trimmed according to the boundary mask of India. The resulting raster layer was then subjected to a quality checking process, whereby only pixels with a quality flag of 0 or 1 were retained for further processing (Fig 1 in S1 File).

**Fig 3:**
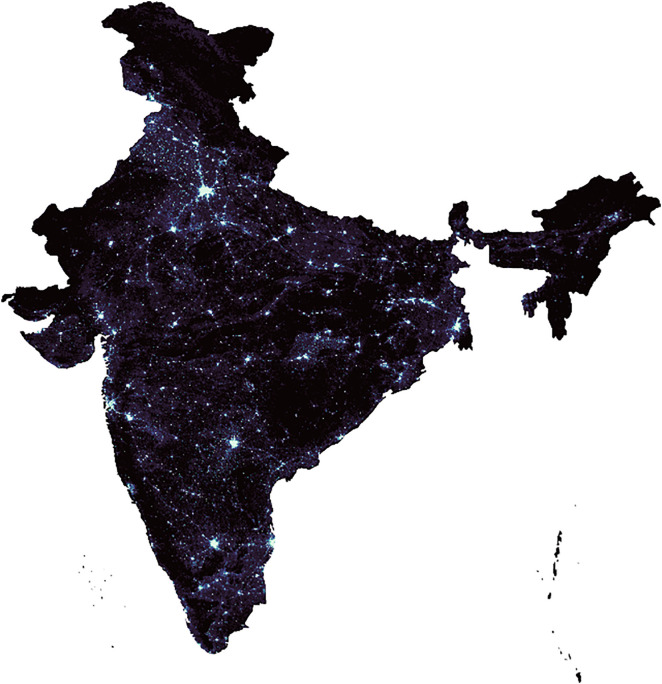
India in 2012.

**Fig 4:**
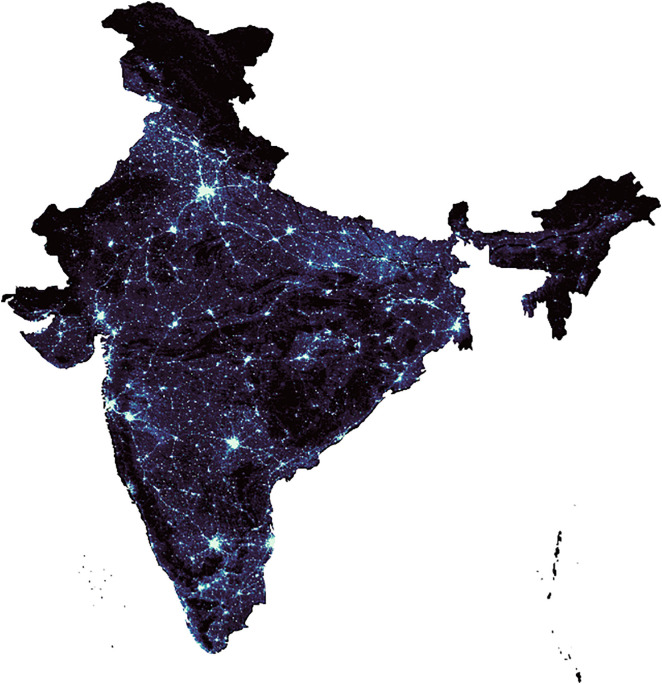
India in 2023.

The significant surge in night time illumination observed in major metropolitan areas of India between 2012 and 2023, as depicted in Figs 3 and 4 and analysed by ISRO, highlights the utility of night lights as a crucial indicator for estimating electricity consumption.

For state wise satellite image extraction, level 1 geographic boundaries of India were utilized to extract state-wise metrics, considering daily and annual temporal variations using VNP46A2/A4, respectively. These datasets were obtained and the appropriate metrics were calculated through GEE and R script, similar to the entire India analysis.

#### 2.1.3. Sum of lights.

Various approaches have been proposed by researchers to extract valuable information from satellite images for electricity consumption estimation. Fehrer [28]} utilized a linear regression model with the TNL index, defined as the sum of radiation values in each pixel normalized by a reference area. Here L represents the radiation and A represents the area. Previously, the TNL index has been utilized among others by Shi et al [29].


$$\begin{array}{c} TNL=\;\sum {\mathrm{\backslash nolimits}}_{k=1}^{K}\left(L_{k}\times \frac{A_{k}}{A_{r}}\right) \end{array}$$ (1)

Similarly, Li et al. [30] employed the SOL index, summing the frequencies of radiance values in each pixel. Here N_i_ represents the frequency of pixels having brightness value B_i_ and B_max_ is the maximum brightness value in a given raster file.


$$\begin{array}{c} SOL=\;\sum {\mathrm{\backslash nolimits}}_{i=1}^{B_{max}}\left(N_{i}\times B_{i}\right) \end{array}$$ (2)

Other studies, like Jing et al. [31], introduced threshold-based approaches to exclude low-value pixels. Additionally, the Compounded Night Light Index (CNLI) was defined in [5,32], accounting for pixel values within a specified range [B_0_, B_max_].


$$\begin{array}{c} CNLI=\;\frac{1}{N_{L}\times B_{max}}\;\times \;\sum {\mathrm{\backslash nolimits}}_{i=B_{0}}^{B_{max}}\left(N_{i}\times B_{i}\right) \end{array}$$ (3)

Among these metrics, SOL has been chosen due to its suitability for quantifying total electricity consumption by summing radiance values within a geographic boundary. VNP46A2/A4 features a radiance range of 0–65535, along with a scale factor of 0.1. Consequently, each pixel value needs to be scaled by 0.1 to obtain the actual radiance value.

**Fig 5:**
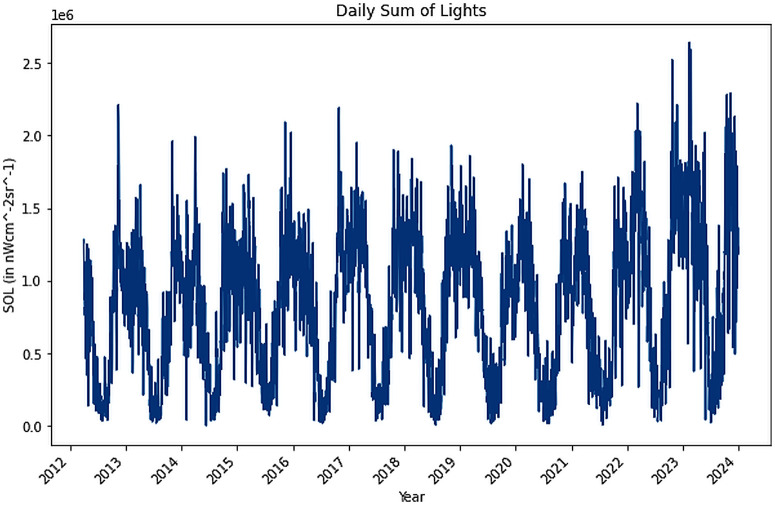
Daily Sum of Lights.

The sum of total radiance values was determined for VNP46A2/A4, with quality pixels of either 0 or 1 only were selected. The pixel values were then scaled by 0.1 and the total sum for the given region was subsequently calculated. Data gaps were addressed by estimating missing values through averaging adjacent days’ values (Fig 5).

**Fig 6:**
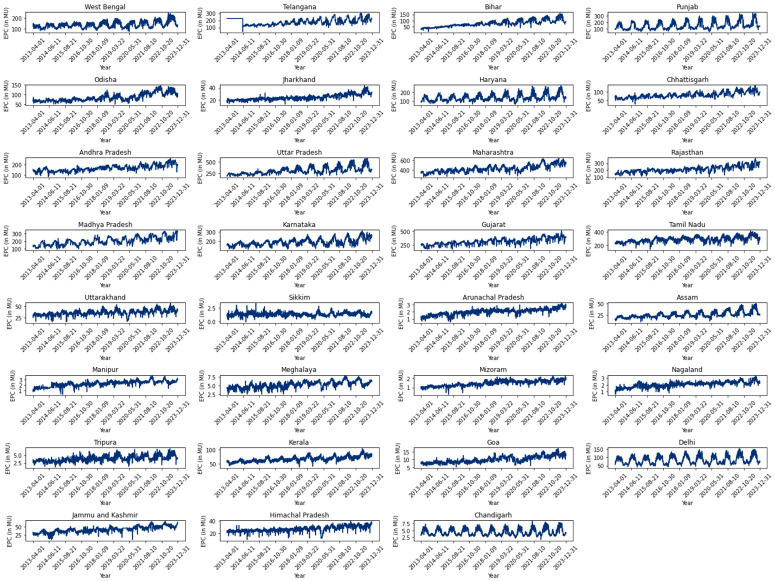
State wise daily Sum of Lights.

State-wise sum of lights was calculated by applying the same methodology as for the entire country, utilizing level 1 geographic boundaries of India. Daily and annual temporal variations were considered using VNP46A2/A4 datasets, and state-wise sum of lights were derived, ensuring consistency with the approach used for the entire India analysis (Fig 6).

### 2.2. Electricity consumption data

**Fig 7:**
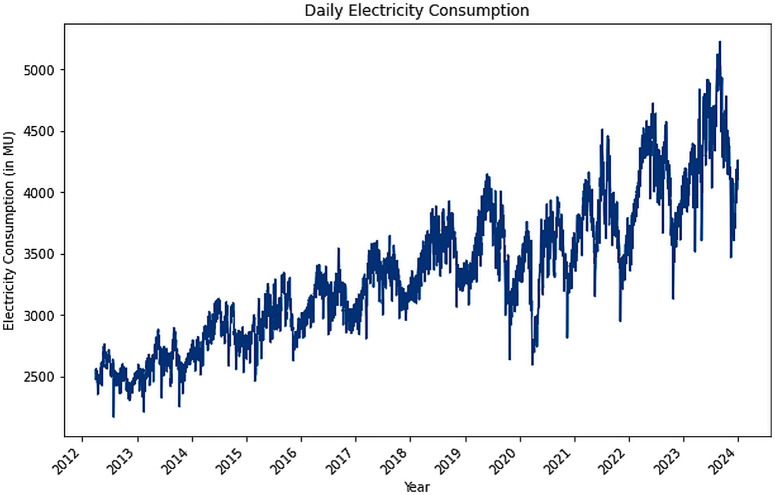
Daily Electric Power Consumption.

The Electric power consumption of India was obtained from Power System Operation Cooperation (POSOCO). The daily power consumption from April 1^st^, 2012 to December 31^st^, 2023 was obtained by downloading monthly PDF reports, and scraping the document to obtain the daily consumption (Fig 7). The figures in these reports are denoted in terms of actual drawal, specifically in MU (1 million kWh).

**Fig 8:**
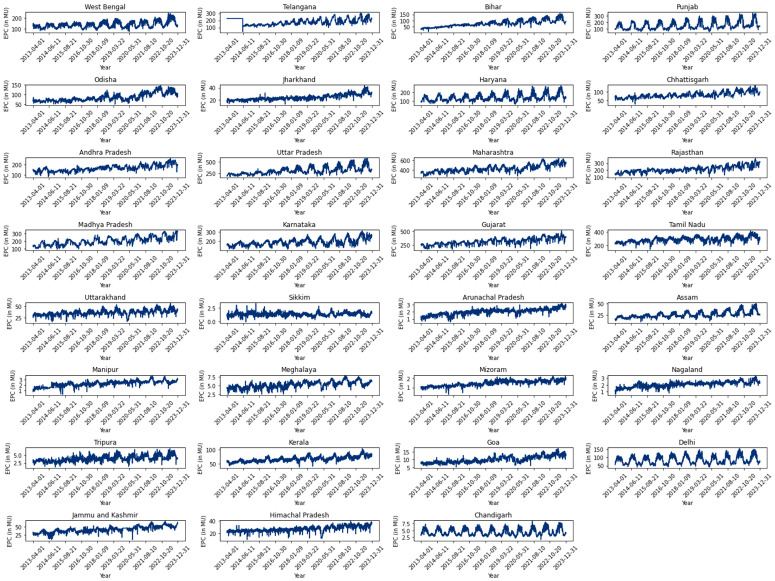
State wise daily Electric Power Consumption.

In addition to extracting the daily power consumption data for India, state-wise daily electricity usage was obtained through web scraping techniques from the POSOCO website. This data spans from April 1^st^, 2013 to December 31^st^, 2023 (Fig 8).

### 2.3. Handling of missing data

#### 2.3.1. Detail the imputation process.

Missing values in the sum of lights (SOL) computation were managed through linear interpolation, where the missing value was approximated as the mean of the previous and next values. It was computed as:


$${SOL}_{t}\;=\;\frac{{SOL}_{t+1}\;+\;{SOL}_{t-1}}{2}$$

This was uniformly applied throughout the dataset to guarantee equal treatment of missing values.

#### 2.3.2. Justify the choice.

The use of linear interpolation was decided based on the following reasons:

- Missing values were few and were randomly distributed across the data set, so it was an effective but easy solution.
- The time series data had high temporal continuity, i.e., nighttime lights’ values between two consecutive days do not differ significantly under standard circumstances.
- More sophisticated techniques (e.g., polynomial interpolation or model-based imputation) were not required with the low rate of missing data and the fairly stable time series.

#### 2.3.3. Assess impact.

In order to assess if the imputed values affected model performance, a sensitivity analysis was performed. The model was trained under two conditions:

- Imputed data (with linear interpolation).
- No imputed data (without missing values).

The findings indicated very slight variations in MAE, RMSE, and R^2^ between both cases, affirming that the method of interpolation introduced no substantial bias. Under such circumstances, linear interpolation was found to be an acceptable option that maintained data integrity while providing a full dataset for model training.

## 3. Proposed system and methodology

This section outlines the study’s aim to integrate night light satellite imagery data, particularly the sum of lights, into forecasting models for electricity consumption. It discusses the utilization of deep learning time series method such as LSTM [5,7,15,17], which excel at capturing complex patterns and dependencies from sequential data. These methods offer advantages over traditional models by effectively handling large datasets, accommodating nonlinear relationships, and capturing temporal dependencies across different time scales. By leveraging advanced neural network architectures, this study seeks to achieve more accurate predictions of electricity consumption trends.

The dataset utilized for this study spans from April 1, 2012, to December 31, 2023, comprising approximately 4,000 data points. This dataset serves as the foundation for evaluating the forecasting models, providing comprehensive insights into electricity consumption patterns over the specified time frame. An 80:20 split of the dataset was implemented to allocate 80% of the data for training and 20% for testing. This division ensures that the model is trained on a sufficiently large dataset while maintaining a separate set of data for unbiased performance evaluation. Before training the models, the dataset underwent normalization using Min-Max scaling. This technique ensures that all feature values fall within the range of 0–1.

Given the time-dependent nature of the data, the split was performed chronologically, ensuring that all data points used for training were from time periods preceding those in the test sets. This approach prevents data leakage and mimics real-world forecasting scenarios.

### 3.1. LSTM

LSTM networks, introduced in 1997 [33], have gained prominence in time series forecasting and sequential data analysis due to their adeptness in capturing long-term dependencies and mitigating the vanishing gradient problem. Comprising memory cells regulated by input, forget, and output gates, LSTMs effectively retain and manipulate information over extended sequences. The input gate determines the influence of new data on the cell state, while the forget gate controls the retention of previous information. The output gate manages the amount of cell state output to the next time step, while the candidate activation function computes a new candidate cell state based on the input and previous hidden state. Through these mechanisms, LSTMs can effectively capture intricate patterns and dependencies in sequential data, making them well-suited for time series forecasting tasks.

In this study LSTM models were assessed by developing a baseline model for univariate electricity forecasting and subsequently incorporating the SOL as a predictor. LSTMs were found to be the most suitable choice for forecasting due to their capability to handle complex dataset patterns without extensive preprocessing, which is often required for statistical models. Unlike machine learning models, LSTM models excel in capturing dataset nuances, making them optimal for accurately predicting outcomes while accommodating dataset intricacies. Although models like TCNs offer powerful alternatives, LSTM’s effectiveness remains robust, particularly when dealing with datasets containing limited data points.

### 3.2. Stacked LSTM

Stacked LSTM networks are a type of RNN architecture consisting of multiple LSTM layers arranged hierarchically. Each layer contains LSTM cells responsible for processing sequential data. The input data traverses through each LSTM layer successively, with each layer generating a hidden state embodying learned features at each time step. This hierarchical arrangement allows stacked LSTMs to learn intricate patterns and relationships, as each layer captures distinct levels of abstraction.

**Fig 9:**
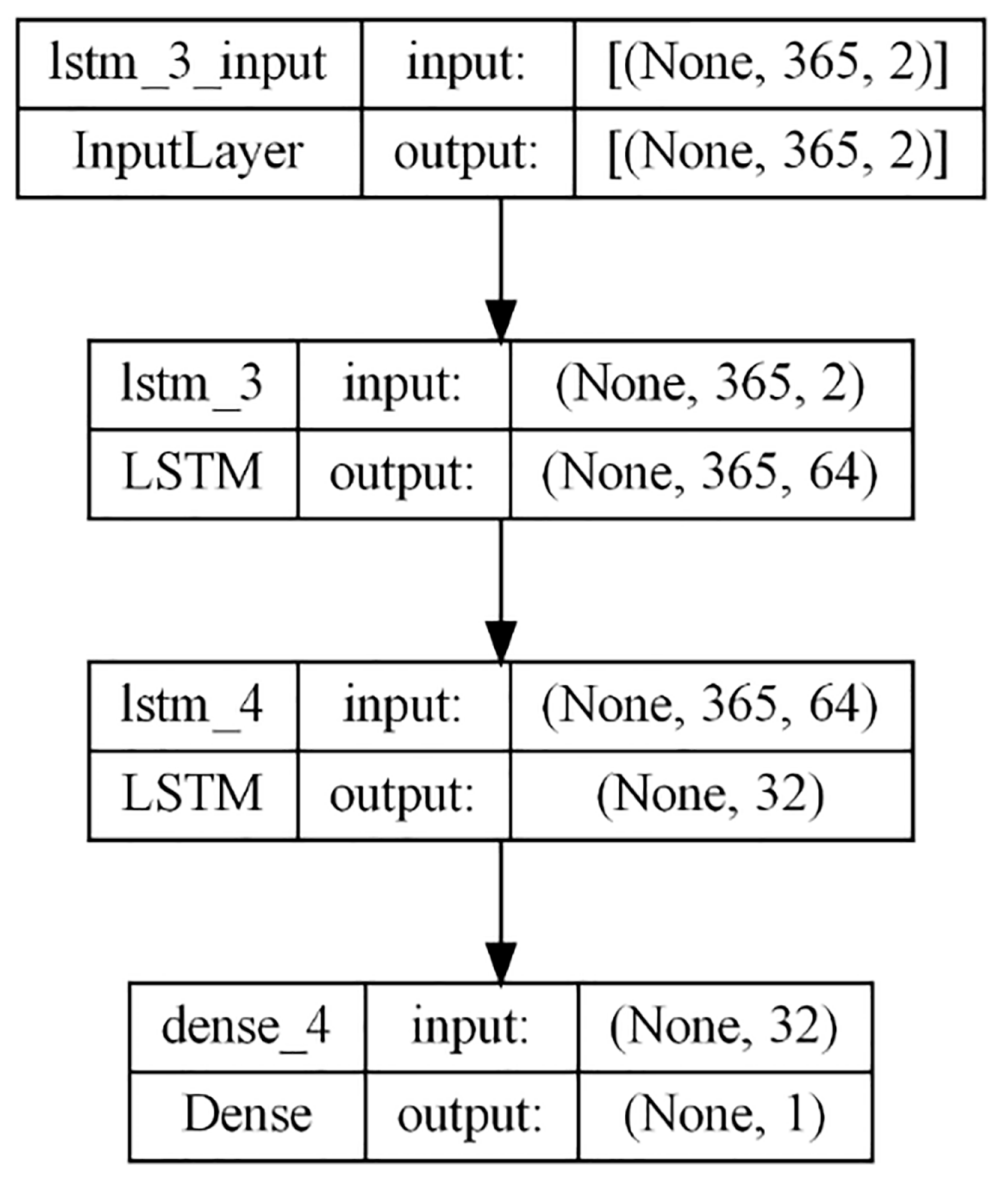
Stacked LSTM.

Additionally, stacking multiple LSTM layers helps mitigate the vanishing gradient problem often encountered in deep neural networks with numerous layers. By propagating the hidden state from one layer to the next, stacked LSTMs facilitate the network in comprehending long-term data dependencies more effectively. In this study, the model architecture comprises two LSTM layers followed by a dense layer (Fig 9), with the hyperparameters optimized for optimal performance, utilizing the Adam optimizer with a learning rate of 0.001 and employing MSE loss and RMSE evaluation metric for assessing model performance.

### 3.3. Bidirectional LSTM

Bidirectional LSTM networks [34] represent an advancement in RNNs, allowing for the processing of input sequences in both forward and backward directions. During the forward pass, the input sequence progresses from the beginning to the end, with the forward LSTM layer generating hidden states that capture dependencies until the current time step. Conversely, in the backward pass, the input sequence is processed from the end to the beginning, and the backward LSTM layer generates hidden states capturing dependencies from future time steps. After both passes, the hidden states from both LSTM layers are combined to create a final representation of the input sequence, incorporating both past and future dependencies.

**Fig 10:**
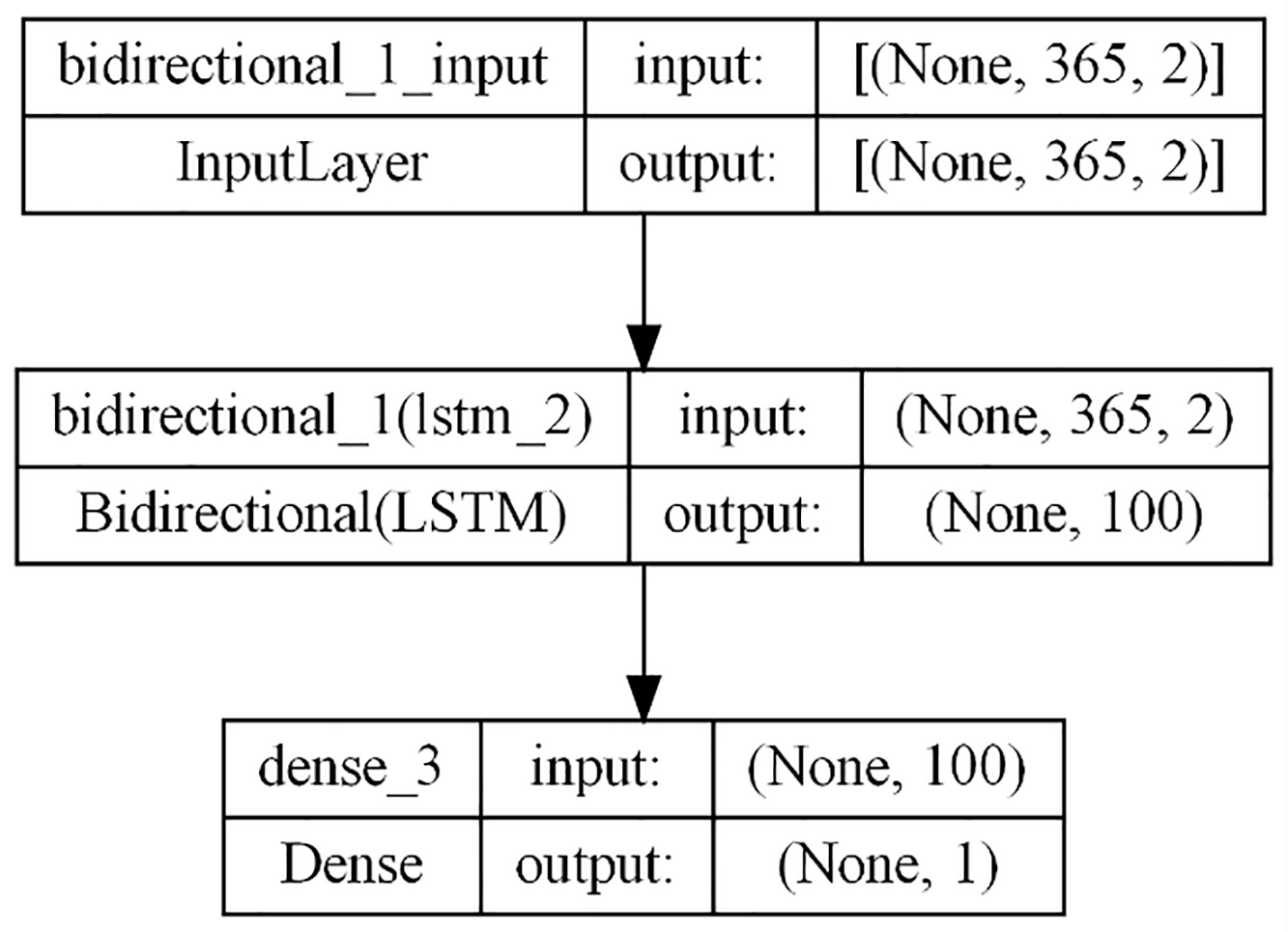
Bidirectional LSTM.

This approach enables bidirectional LSTMs to leverage information from both preceding and succeeding time steps and thus capture extensive dependencies in sequential data, leading to enhanced predictive accuracy or classification capabilities. The model utilized in this study employs a single Bidirectional LSTM layer followed by a dense layer (Fig 10). The hyperparameters were optimized, utilizing the Adam optimizer with a learning rate of 0.001, and the model’s performance was evaluated using MSE loss and RMSE evaluation metric.

### 3.4. CNN-LSTM

The CNN-LSTM model [35] represents a hybrid architecture that combines the strengths of CNNs and LSTM networks. In the CNN-LSTM architecture, CNNs process the input time series data to extract spatial patterns, which are then fed into LSTM layers to capture temporal dependencies. This integration enables the model to effectively capture both short-term and long-term patterns in time series data, leading to enhanced forecast accuracy.

**Fig 11:**
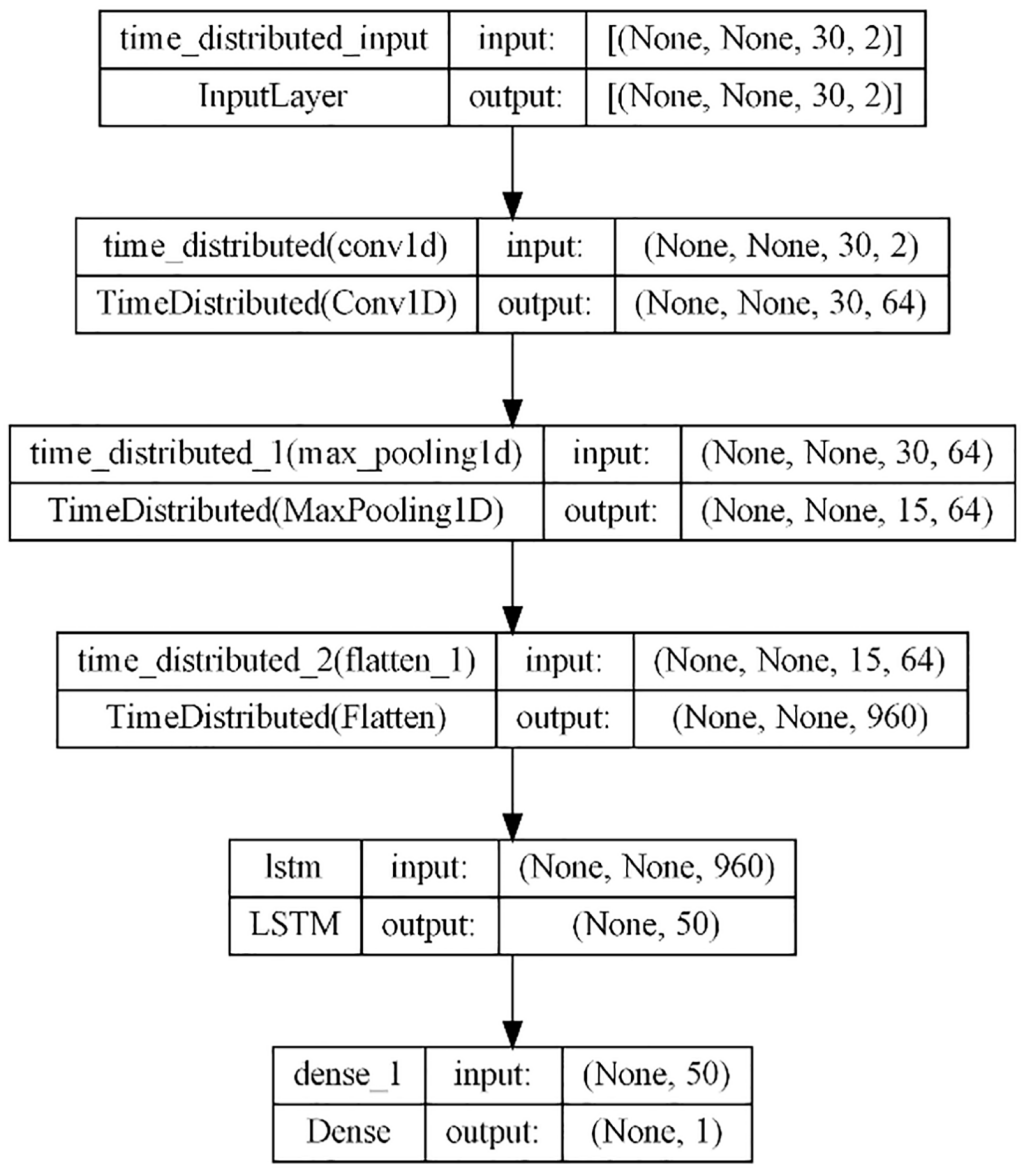
CNN-LSTM.

The model employed in this study comprises a single Convolutional layer followed by a MaxPooling layer, which is then flattened and forwarded to an LSTM layer and finally to a dense layer (Fig 11). The number of features is set at 1 for univariate forecasting and 2 when SOL is included. The model’s hyperparameters, including the Adam optimizer with a learning rate of 0.001, were optimized to ensure optimal performance. Loss assessment was conducted using MSE, while RMSE served as the chosen evaluation metric, ensuring accurate evaluation of the model’s forecasting capabilities.

### 3.5. ConvLSTM

Convolutional LSTM cells represent a novel architecture [36] that combines the memory and hidden states of traditional LSTM cells with convolutional operations, allowing them to operate directly on input tensors and capture spatial information. Unlike traditional LSTMs, ConvLSTM cells compute input, forget, and output gates, as well as the candidate memory cell state, using convolutional operations, which enables them to learn spatially varying gating mechanisms and effectively capture spatial dependencies within the input data.

**Fig 12:**
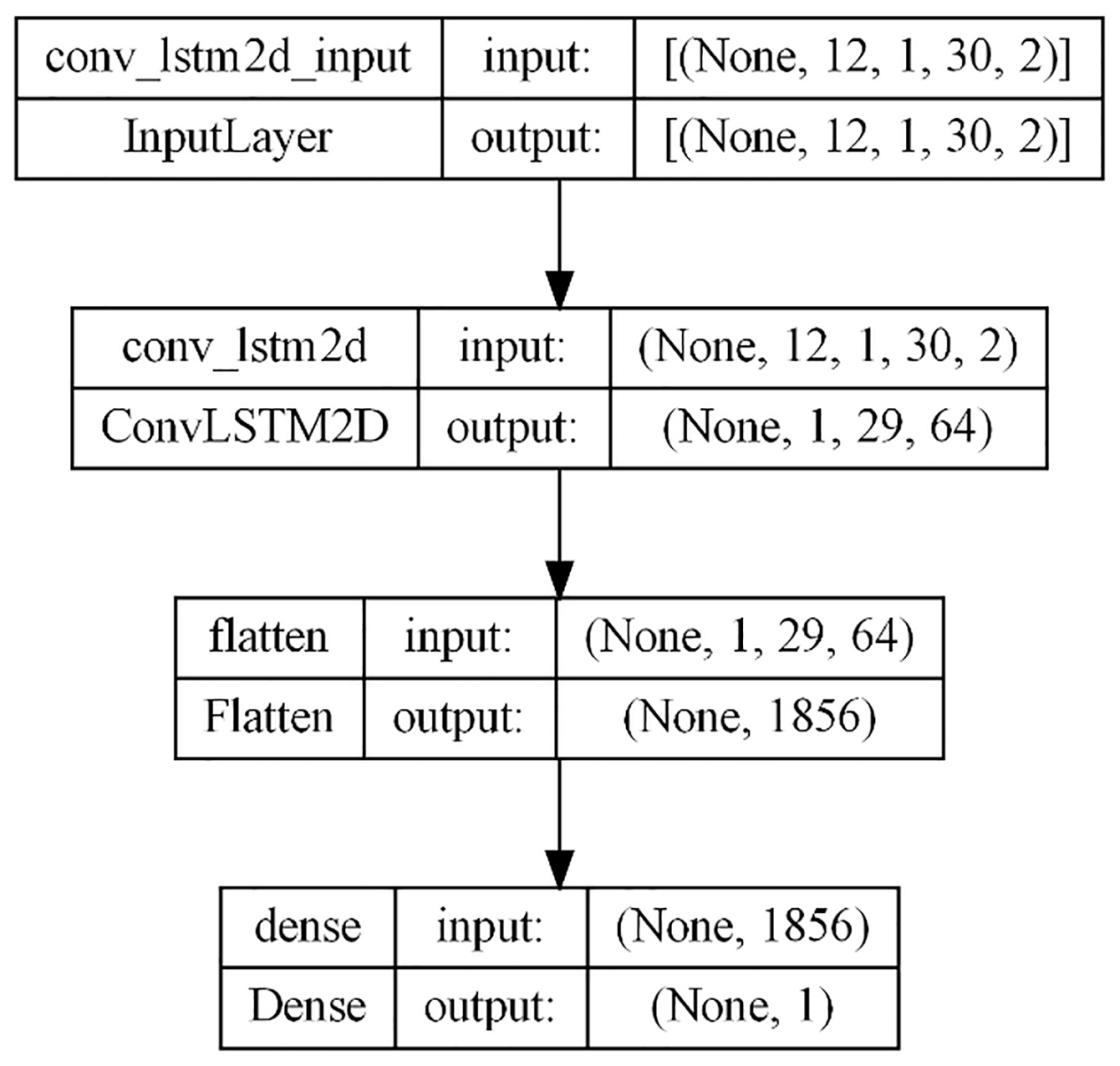
ConvLSTM.

ConvLSTM networks can be organized hierarchically, with each layer consisting of multiple ConvLSTM cells, enabling the network to learn the abstract representations of the input data, with lower layers focusing on local spatial patterns and higher layers capturing global patterns. In the model utilized for this study, a single Convolutional layer is followed by a MaxPooling layer, which is then flattened and passed into an LSTM layer before being fed into a dense layer (Fig 12). The number of features set at 1 for univariate forecasting and 2 for multivariate forecasting with the inclusion of the SOL. Hyperparameter tuning involved selecting the Adam optimizer with a learning rate of 0.001, while loss evaluation utilized the MSE, and the RMSE was chosen as the evaluation metric to assess the model’s forecasting performance accurately.

### 3.6. State wise analysis

To enhance the accuracy of EPC estimation, a state-wise approach was adopted, leveraging NTL data to capture spatial variations for more precise forecasts. Initially, annual SOL was computed for each state, and trend analysis was conducted to identify patterns. Mean, standard deviation, and slope of these trends were computed, followed by k-means clustering to group states based on their consumption patterns. The silhouette score method and elbow method were then employed to determine the optimal number of clusters. The silhouette score assessed cluster cohesion and separation, while the elbow method identified the point where the decrease in Within Cluster Sum of Squares rate slowed down, indicating the optimal number of clusters. These methodologies aimed to identify the most appropriate clustering arrangement to capture distinct consumption patterns across various states.


$$S(i)=\frac{b(i)-a(i)}{\text{max}(\{a(i),b(i)\})}$$ (4)

$$\begin{array}{c} WCSS=\;\sum {\mathrm{\backslash nolimits}}_{i=1}^{k}\sum {\mathrm{\backslash nolimits}}_{x\in C_{i}}{\left|\left|x-\;\mu_{i}\right|\right|}^{2} \end{array}$$ (5)

Subsequently, states were grouped into corresponding clusters, and various models identified earlier was used to fit and forecast electricity consumption for each group separately. Daily SOL for each state was acquired, and the total EPC and SOL for each cluster group were computed. The optimal model was then applied to fit and forecast future EPC for each aggregated group, allowing for more accurate forecasting tailored to the specific consumption patterns observed within each cluster (Fig 2 in S1 File). Overall, this state-wise clustering approach enabled the incorporation of spatial variations, enhancing the precision of electricity consumption estimation across different regions.

## 4. Results and discussion

### 4.1. Univariate electricity forecast

**Fig 13:**
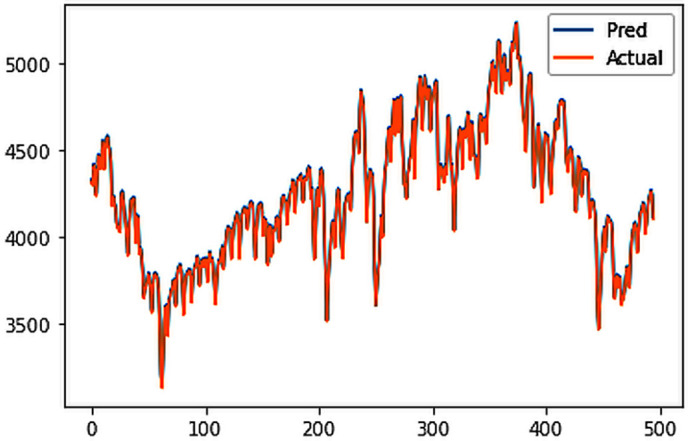
Stacked LSTM.

**Fig 14:**
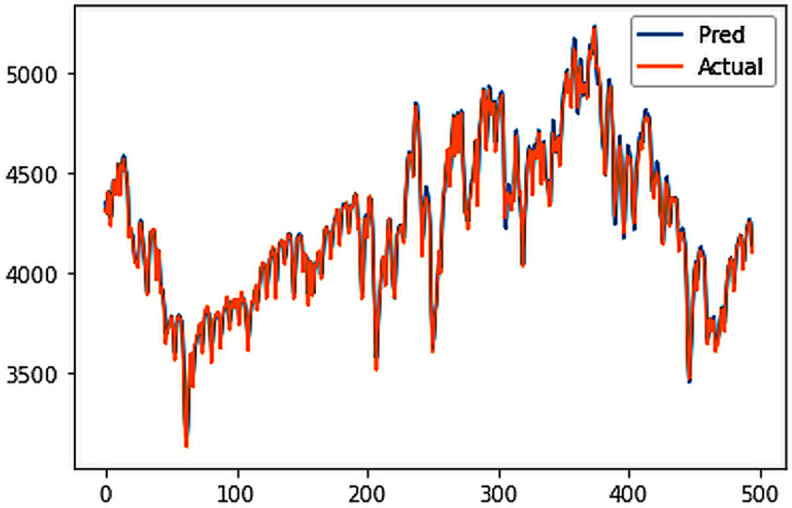
Bidirectional LSTM.

**Fig 15:**
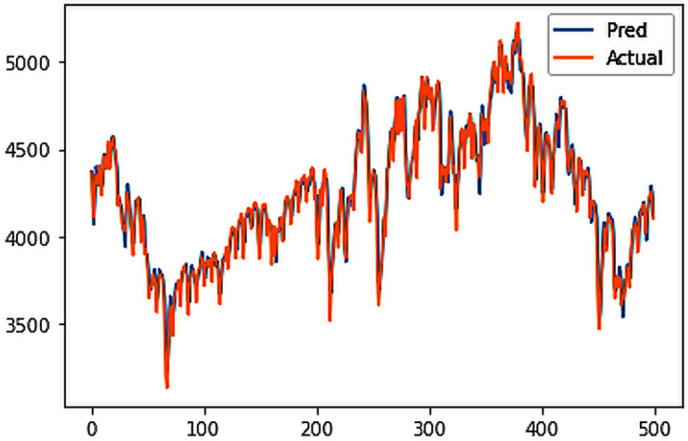
CNN-LSTM.

**Fig 16:**
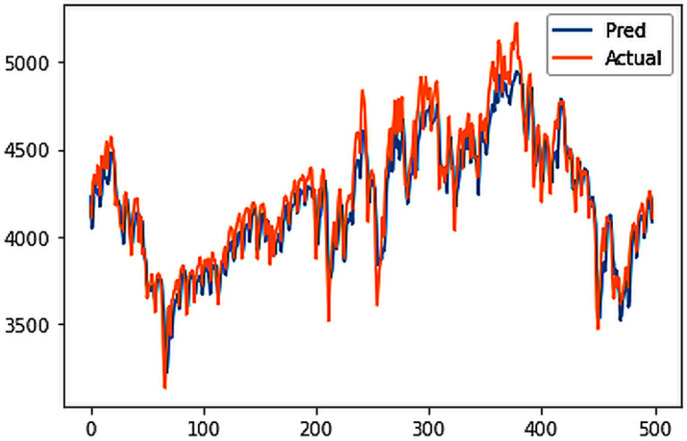
ConvLSTM.

Different LSTM model variants were utilized to model univariate electricity consumption data for the entire country. These findings as shown in Figs 13–16 highlight the superior performance of the stacked LSTM model, which demonstrated the lowest RMSE value of 12.066 MU and effectively captured underlying consumption patterns. Conversely, more complex models exhibited overfitting issues, leading to higher RMSE values. Therefore, for this dataset, the stacked LSTM model emerged as the most suitable choice for forecasting electricity consumption patterns.

### 4.2. Multivariate electricity forecasts

**Fig 17:**
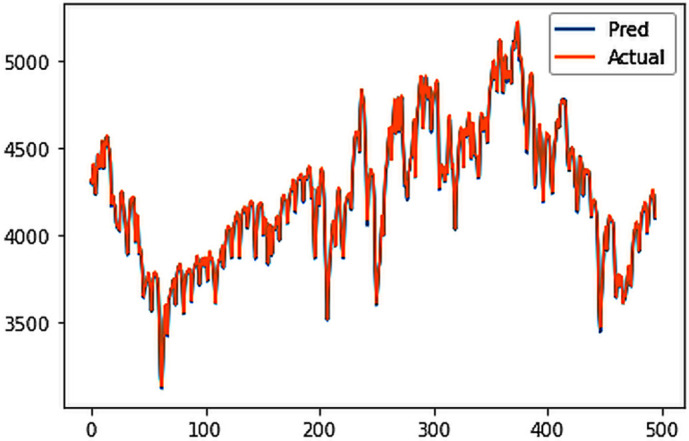
Stacked LSTM.

**Fig 18:**
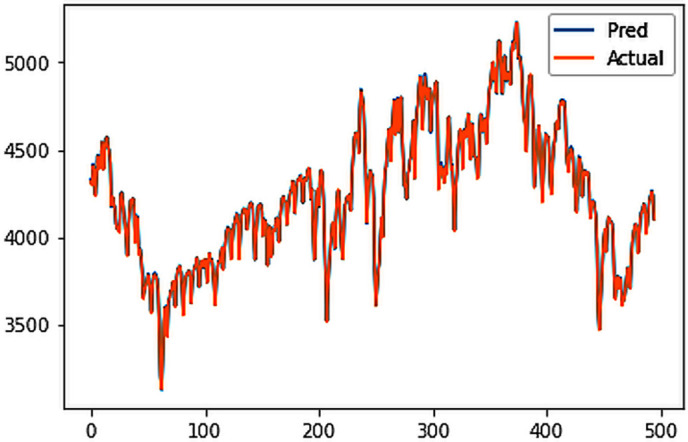
Bidirectional LSTM.

**Fig 19:**
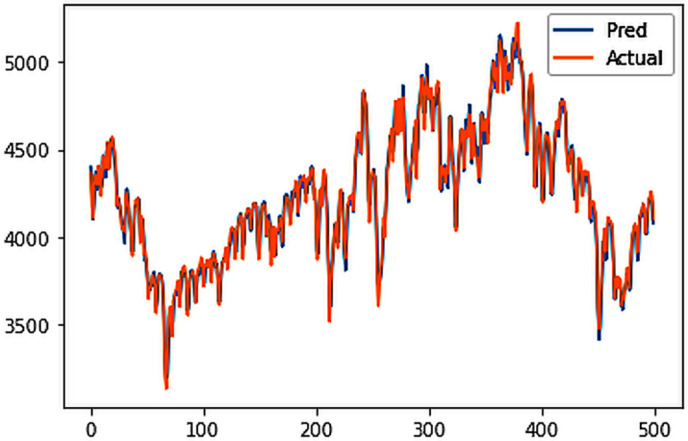
CNN-LSTM.

**Fig 20:**
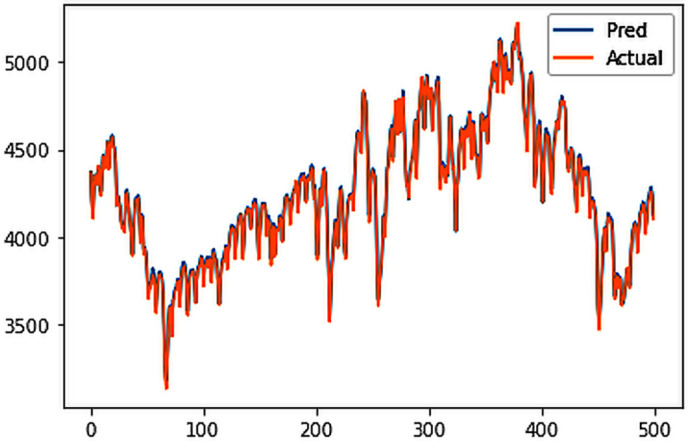
ConvLSTM.

The analysis was extended to include multivariate forecasting, incorporating SOL as an additional predictor variable. These results as shown in Figs 17–20 indicate that multivariate analysis outperforms univariate analysis, with the bidirectional LSTM model achieving the lowest RMSE of 6.045 MU among all models. Conversely, the CNN-LSTM hybrid model demonstrated potential overfitting, leading to a higher RMSE. In general, all the different variations of LSTM models exhibited smoother and better curves when compared to univariate modelling.

The bidirectional LSTM model’s capability to process input sequences both forward and backward complements its effectiveness in capturing intricate patterns, particularly in tasks like EPC forecasting when coupled with SOL data. By incorporating information from both past and future time steps, it gains a comprehensive understanding of the underlying trends in EPC and their correlations with SOL values. This holistic view allows the model to anticipate future consumption patterns while leveraging insights from historical data, thereby enhancing its accuracy in forecasting electricity demands.

Moreover, the comparison of RMSE values in the Table 1 highlights the superiority of multivariate forecasting over univariate forecasting. The inclusion of the sum of lights alongside electricity consumption results in significantly reduced RMSE values, indicating a robust correlation between night lights and power usage. This underscores the importance of incorporating additional variables like sum of lights for more accurate and reliable electricity consumption forecasts.

**Table 1:** Univariate and multivariate results.

Model	Univariate				Multivariate			
	Training Loss	MAE	Testing RMSE (in MU)	Testing R^2^	Training Loss	MAE	Testing RMSE (in MU)	Testing R^2^
**Stacked LSTM**	3.1722e-06	**2.41**	**3.82**	**0.90**	4.5499e-06	6.49	6.82	0.90
**Bidirectional LSTM**	1.1944e-04	21.10	27.66	0.85	9.7537e-06	**2.16**	**2.83**	**0.94**
**CNN-LSTM**	4.0648e-04	45.90	67.24	0.75	3.1658e-04	35.98	46.83	0.76
**ConvLSTM**	9.7672e-04	96.50	118.39	0.69	1.8147e-05	8.81	10.70	0.82

### 4.3. Statistical significance tests

#### 4.3.1. Wilcoxon signed-rank test.

The Wilcoxon signed-rank test is a non-parametric test that is applied in paired data (such as Univariate vs. Multivariate models) where we are not assuming normality.

It ranks absolute differences between pairs, recording if each difference is positive or negative, and subsequently computes a test statistic to determine whether the differences are significant or not.

#### 4.3.2. Interpretation.

p-value = 0.25, which is higher than 0.05.

**Fig 21:**
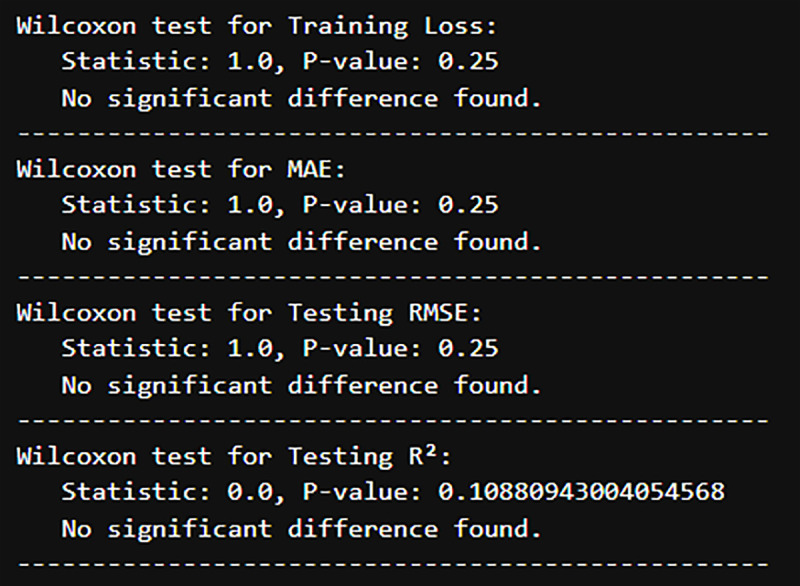
Wilcoxon Testing for each Metric.

This indicates there is little to no difference between Univariate and Multivariate models in MAE. The same applies to Training Loss, RMSE, and R^2^, producing the same results. The results are shown in Fig 21.

#### 4.3.3. Results.

##### 4.3.3.1. Friedman test for model comparisons:

The Friedman test is a non-parametric statistical test applied to compare more than two related groups (e.g., varying models) when normality is not an assumption. It is especially effective to compare models across several conditions, for example, Univariate and Multivariate situations.

##### 4.3.3.2. Interpretation:

**Fig 22:**
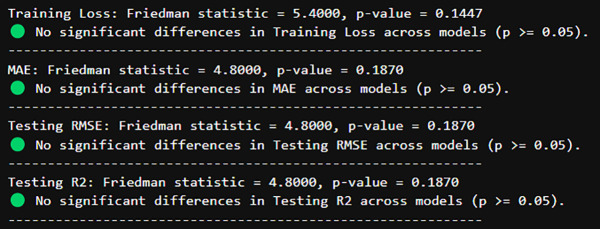
Friedman Testing for each Metric.

p-value < 0.05 → There is a significant difference among models. This implies that one or more of the models perform considerably better or worse than others on a given metric.

p-value ≥ 0.05 → No significant difference between models. This suggests that all models are equally good, and any differences seen are most likely due to randomness and not systematic differences. The results are shown in Fig 22.

### 4.3.3.3. Results: 4.4. Forecasting electric power consumption

The preceding univariate and multivariate analyses focused on single-step forecasts and assessed the model’s performance in fitting unseen testing data. In contrast, this section utilizes the complete dataset to generate new forecasts, extending beyond the scope of the model’s prior exposure. The application of various forecasting models, particularly the CNN-LSTM hybrid model, proved highly effective in generating multi-step forecasts for future electricity demands, exhibiting remarkable precision even for extended periods like the subsequent 60 days. Through rigorous testing, the CNN-LSTM model demonstrated superior accuracy, with an RMSE of **109.647 MU** for the forecasted data, which pertained to January and February 2024. This model’s ability to efficiently capture intricate patterns essential for multi-step forecasting, while retaining past information for accurate future predictions, underscores its utility in energy planning and management. Additionally, while other models like stacked LSTM and Bidirectional LSTM excel in single-step forecasts, the CNN-LSTM hybrid model outperforms them in the complexity of multi-step forecasting.

### 4.5. State wise electric power consumption

#### 4.5.1. K-means clustering.

**Fig 23:**
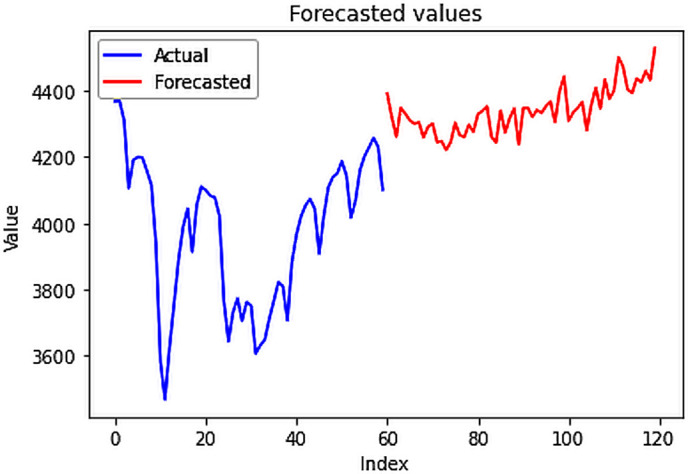
Multi-step forecasted values.

**Fig 24:**
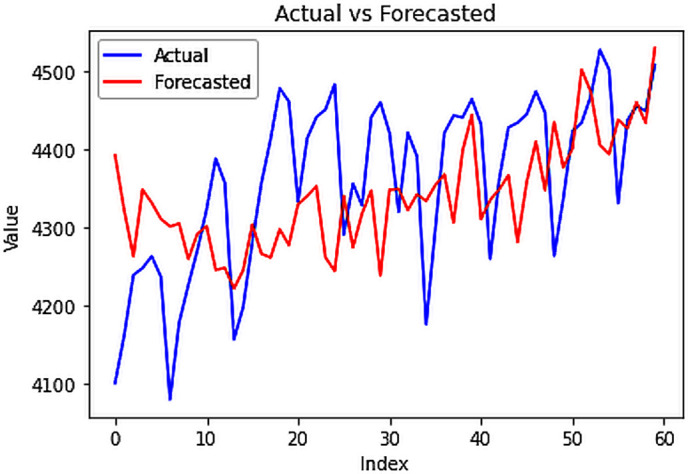
Actual vs Forecasted values.

**Fig 25:**
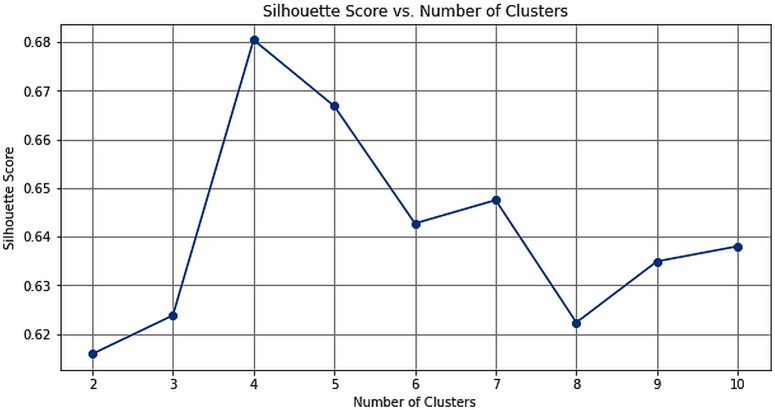
Silhouette Score.

The analysis of the annual SOL across Indian states involved extracting time series data and examining their trends through mean, standard deviation, and slope calculations (Fig 25). These metrics provided insights into the variations in nighttime illumination across different regions. Utilizing k-means clustering, various techniques like silhouette score and the elbow method were employed to determine the optimal number of clusters for grouping the states. The silhouette score peaked at 4 clusters (Fig 23), which aligns with the elbow point indicated by the elbow curve analysis (Fig 24). This meticulous analysis allowed for the effective categorization of states based on their annual SOL, facilitating a deeper understanding of spatial variations in nighttime illumination patterns.

**Fig 26:**
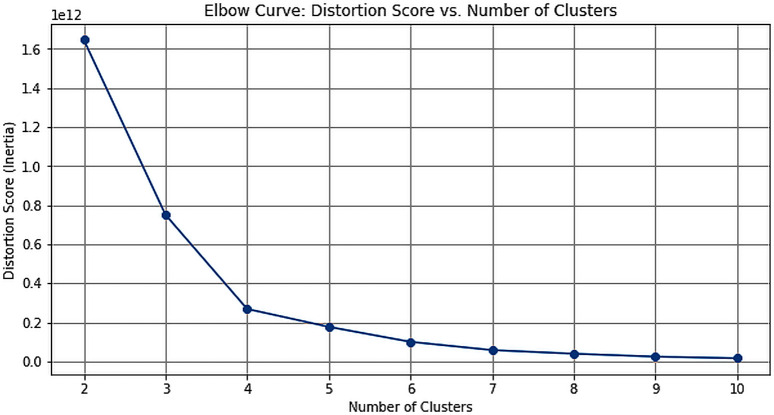
Elbow Curve.

**Fig 27:**
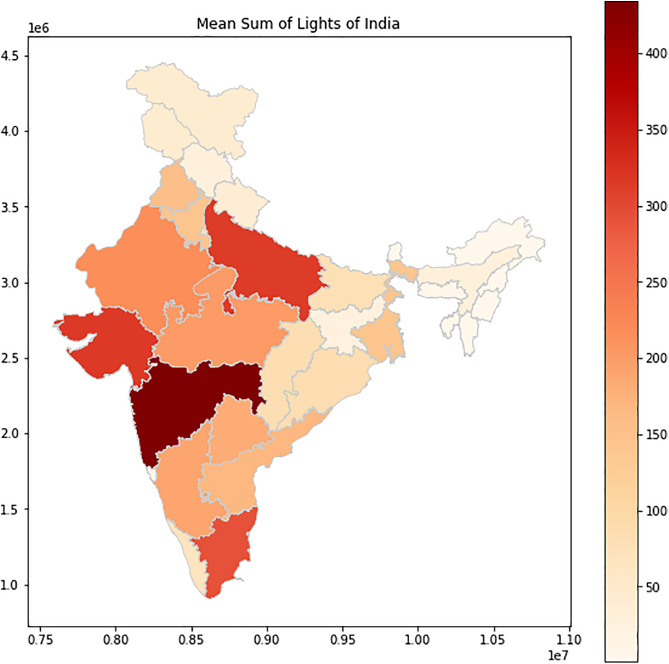
Mean Sum of Lights of India – State-wise.

**Fig 28:**
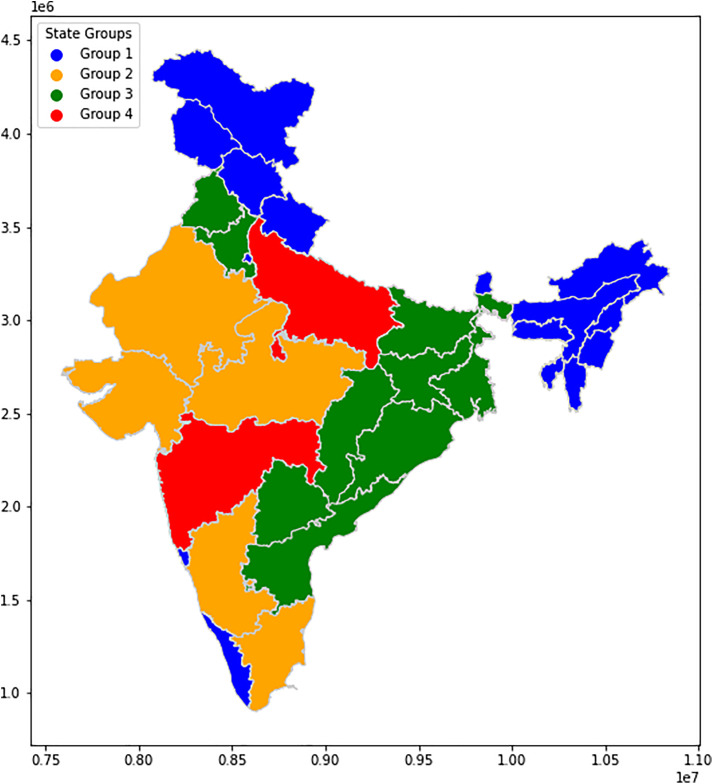
State-wise cluster groups.

The clustering results revealed distinct groupings of states based on their nighttime illumination trends, providing valuable insights into regional differences in electricity consumption patterns (Figs 26–28). The study does not include Union Territories solely focussing only on state-wise patterns in nighttime illumination across India.

**Cluster 1:** Uttarakhand, Tripura, Sikkim, Nagaland, Delhi, Mizoram, Meghalaya, Manipur, Kerala, Jammu Kashmir, Himachal Pradesh, Goa, Chandigarh, Assam, and Arunachal Pradesh

**Cluster 2:** Tamil Nadu, Rajasthan, Madhya Pradesh, Karnataka, Gujarat

**Cluster 3:** West Bengal, Telangana, Punjab, Odisha, Jharkhand, Haryana, Chhattisgarh, Bihar, Andhra Pradesh

**Cluster 4:** Maharashtra, Uttar Pradesh

#### 4.5.2. Model evaluation.

**Fig 29:**
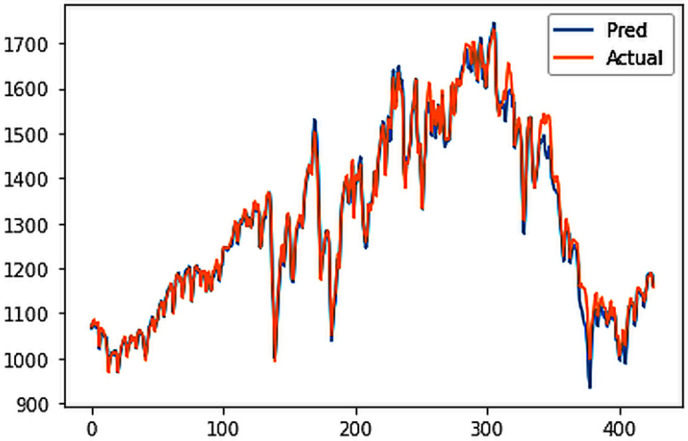
Cluster group 1.

**Fig 30:**
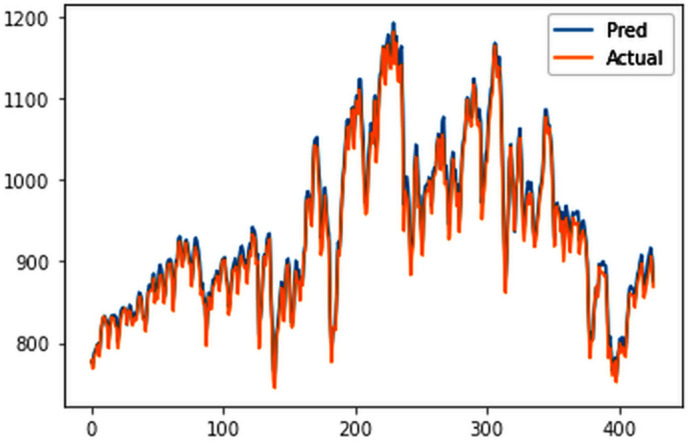
Cluster group 2.

**Fig 31:**
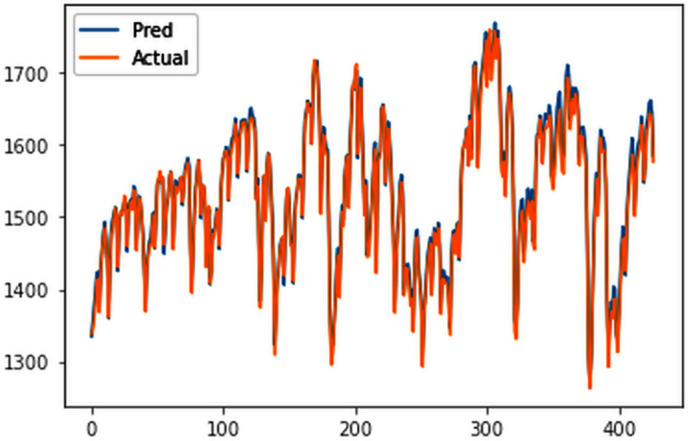
Cluster group 3.

**Fig 32:**
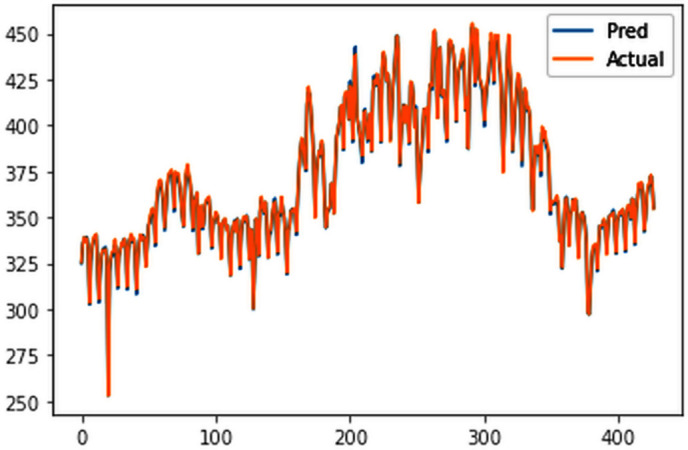
Cluster group 4.

The ConvLSTM model effectively explained variances in cluster groups, with individual training conducted for each group based on their spatial variations in electricity consumption. Results surpassed those of multivariate analysis for the entire country, with all group-specific RMSEs falling below 20 MU, highlighting the efficacy of incorporating spatial variations for enhanced forecasting accuracy. The RMSE values for the four clusters are 18.84 MU, 10.3 MU, 9.83 MU, and 1.43 MU for cluster 1 (Fig 29), cluster 2 (Fig 30), cluster 3 (Fig 31) and cluster 4 (Fig 32) respectively. Cluster-specific RMSE values indicated satisfactory fits for all clusters, supported by visual comparisons between predicted and actual values. Overall, this analysis underscores the value of night light images in understanding spatial fluctuations in electricity consumption and improving forecasting accuracy.

#### 4.5.3. Electricity forecast of individual cluster groups.

**Fig 33:**
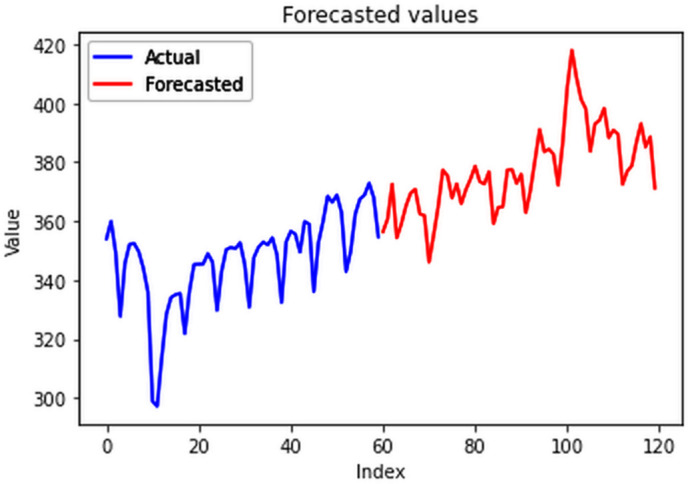
Cluster group 1.

**Fig 34:**
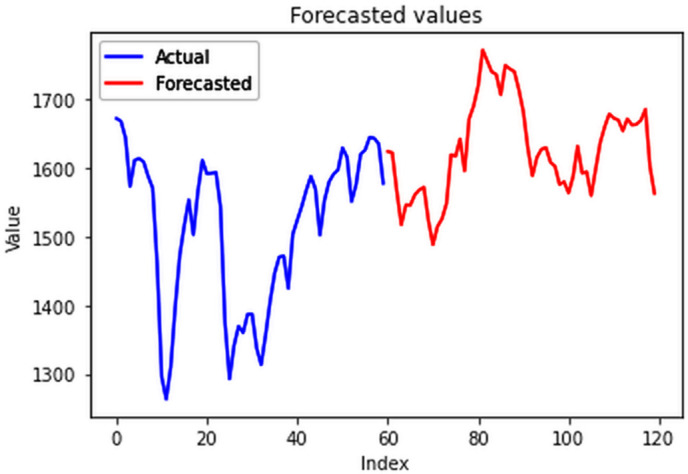
Cluster group 2.

**Fig 35:**
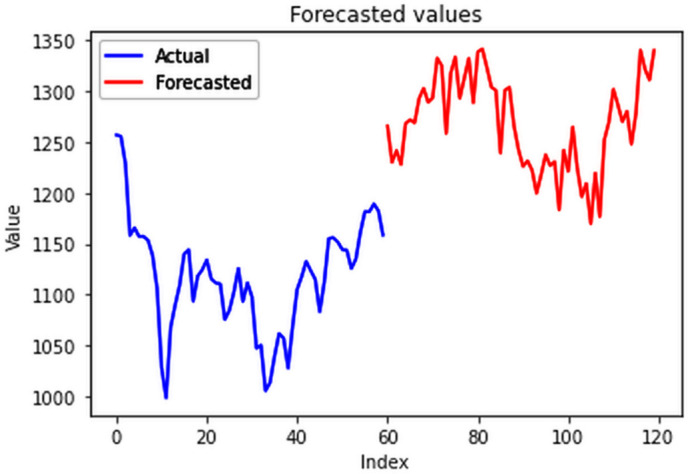
Cluster group 3.

**Fig 36:**
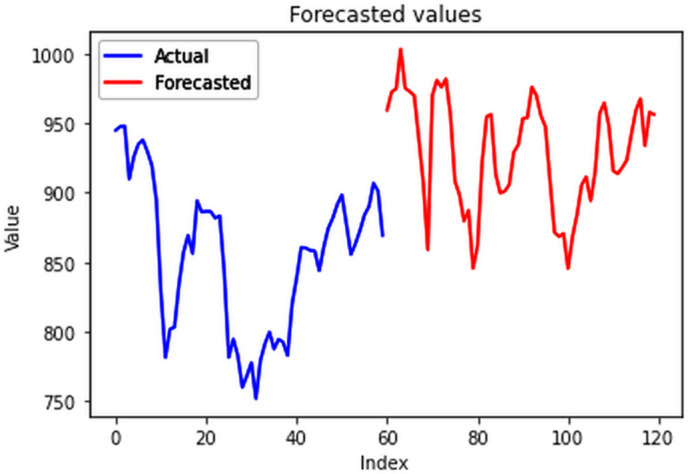
Cluster group 4.

The forecasts (Figs 33–36) for state wise cluster groups were conducted utilizing the ConvLSTM model. This model effectively captured spatial dependencies and temporal variations, and was able to make accurate predictions for each clustered group. By training the ConvLSTM model on the specific characteristics of each state wise cluster group, tailored forecasting solutions were developed to address the unique consumption patterns observed within each cluster, thereby enhancing the precision and reliability of the predictive outcomes.

**Fig 37:**
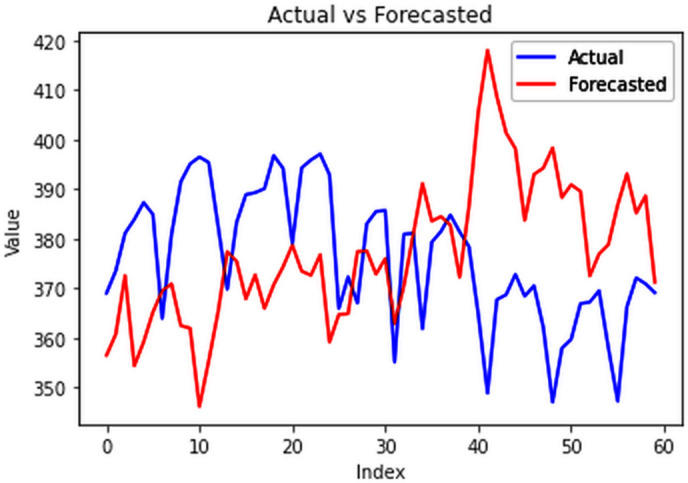
Cluster group 1.

**Fig 38:**
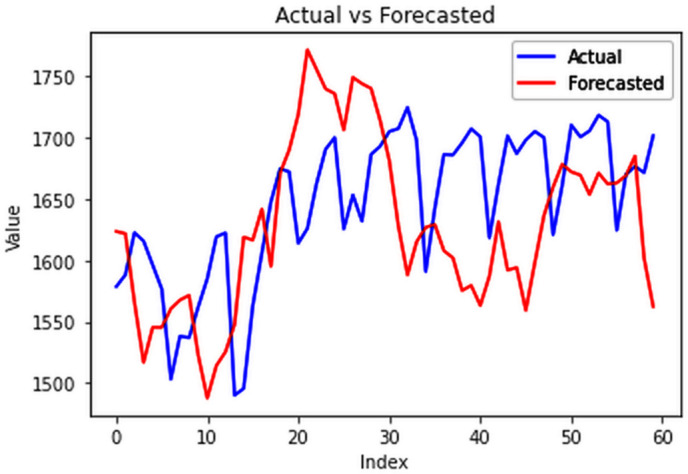
Cluster group 2.

**Fig 39:**
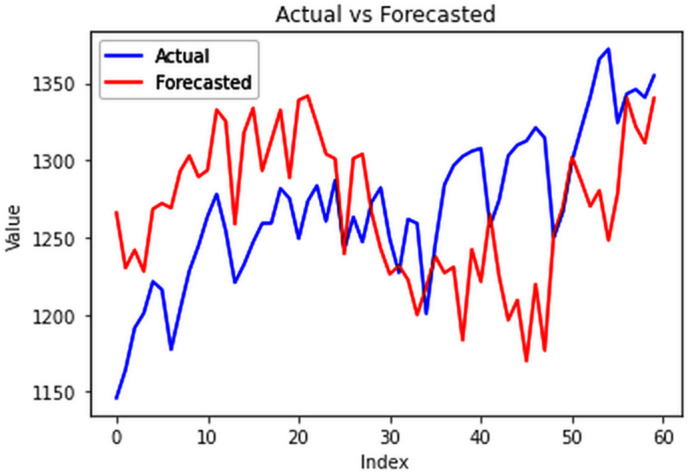
Cluster group 3.

**Fig 40:**
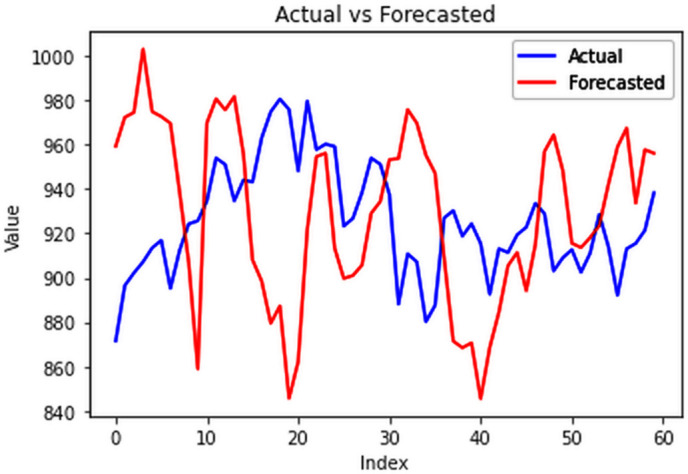
Cluster group 4.

The forecasted data was then compared with the actual EPC values of January and February, 2024 and the evaluation of forecasting performance across various clusters highlights the efficiency of ConvLSTM model. All the clusters had a reasonable RMSE, while all the MAPE values were less than 10% (Table 2). A MAPE value less than 10% indicates accurate forecasting results as shown in Figs 37–40. These results underscore the ConvLSTM model’s effectiveness in capturing diverse consumption patterns within different clusters, enabling more informed decision-making in energy management and planning.

**Table 2:** State-wise forecast results.

	RMSE (in MU)	MAPE
**Cluster 1**	24.36	5.29
**Cluster 2**	76.19	3.97
**Cluster 3**	64.78	4.21
**Cluster 4**	51.98	4.69

### 4.6. Residual analysis

#### 4.6.1. Residual analysis for model evaluation.

For the purpose of ensuring the accuracy of our time series forecasting models, we carried out a residual analysis to check for stationarity. The Augmented Dickey-Fuller (ADF) test was utilized to check if the residuals are stationary, an important requirement for testing the assumptions of time series modeling. The ADF test statistics and p-values for each model are reported in Table 3. The Residual Analysis of Conv-LSTM is shown in Figs 41–44. The Residual Analysis of CNN-LSTM is shown in Figs 45–48. The Residual Analysis of Bidirectional-LSTM is shown in Figs 49–52. The Residual Analysis of Stacked LSTM is shown in Figs 53–56.

**Table 3:** Model -wise ADF test results.

Model	ADF Statistic	p-value	Conclusion
ConvLSTM	−6.1551	7.40e-08	Residuals are stationary
CNN-LSTM	−4.2373	0.00057	Residuals are stationary
Bidirectional LSTM	−4.1331	0.00085	Residuals are stationary
Stacked LSTM	−2.7919	0.05944	Residuals are borderline stationary

**Fig 41:**
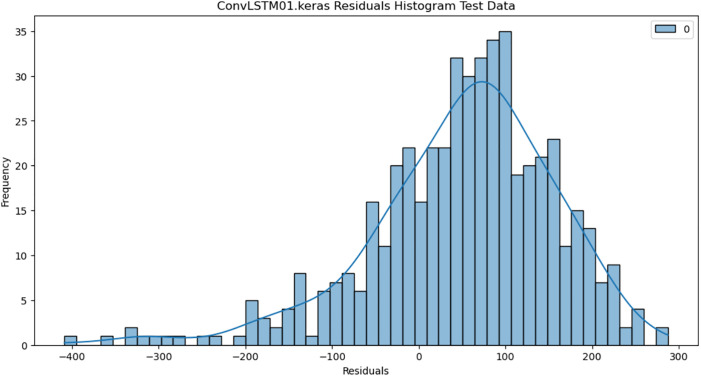
Residuals Histogram.

**Fig 42:**
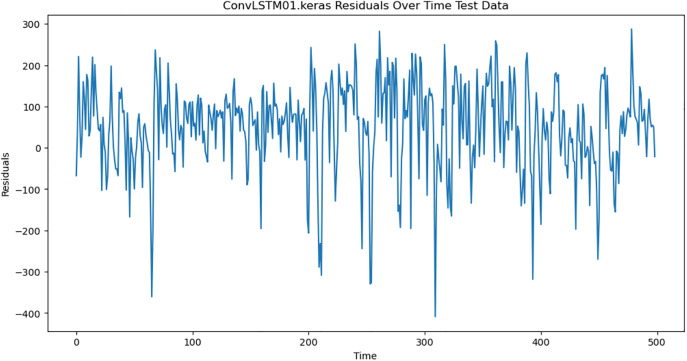
Residuals Over Time.

**Fig 43:**
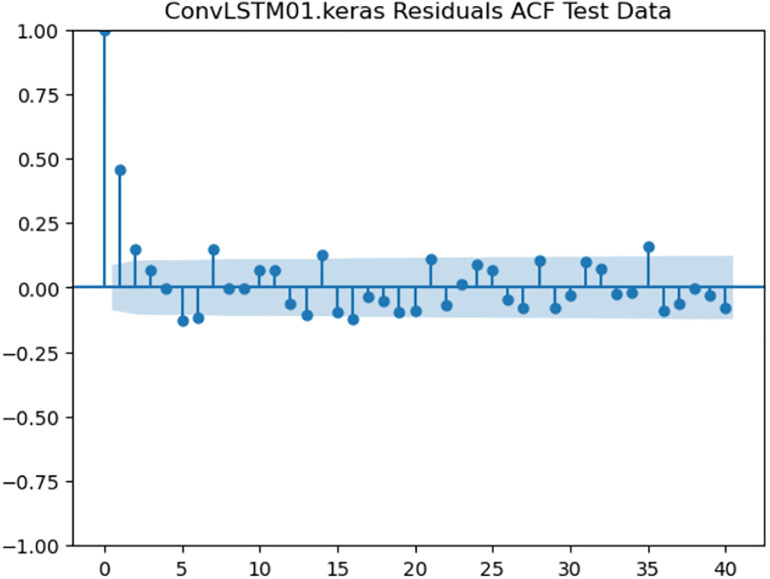
Residuals ACF Data.

**Fig 44:**
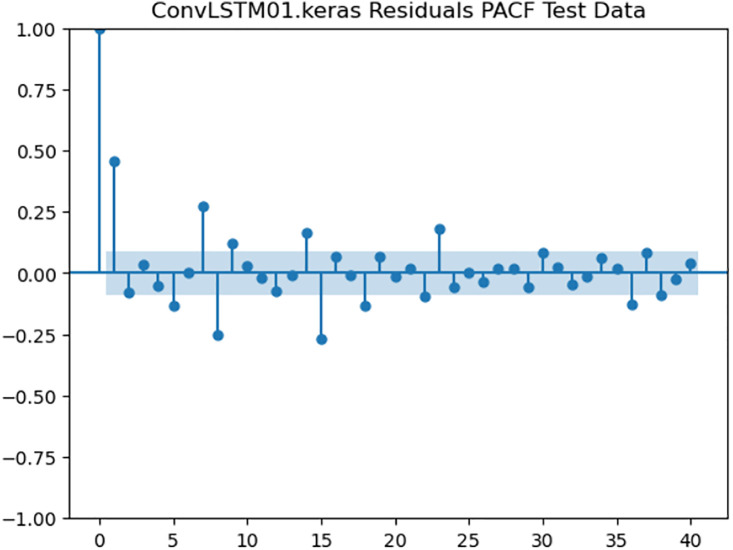
Residuals PACF Data.

**Fig 45:**
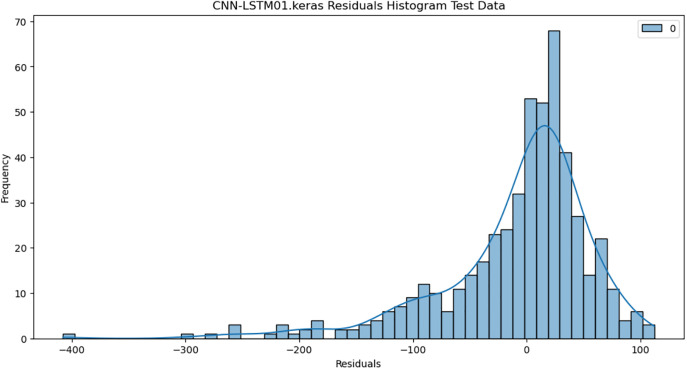
Residuals Histogram.

**Fig 46:**
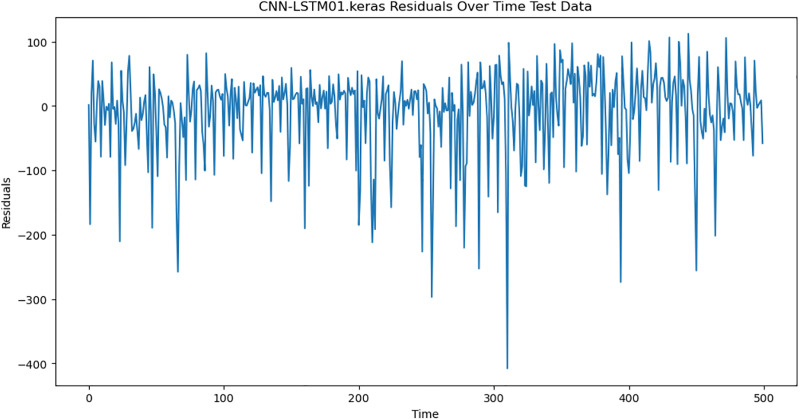
Residuals Over Time.

**Fig 47:**
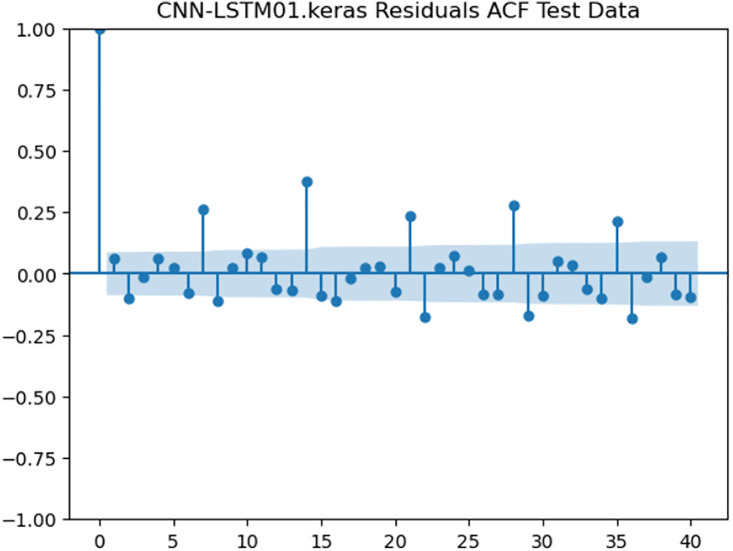
Residuals ACF Data.

**Fig 48:**
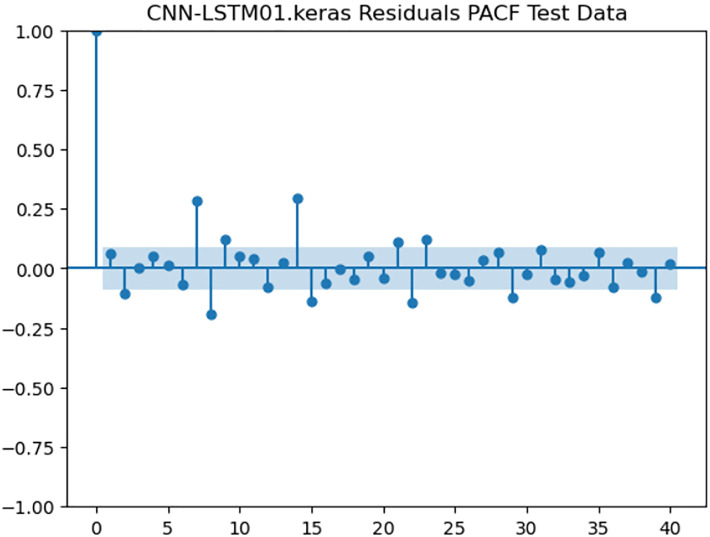
Residuals PACF Data.

**Fig 49:**
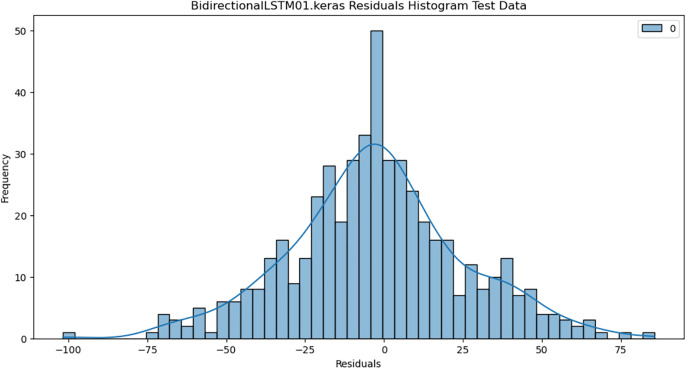
Residuals Histogram.

**Fig 50:**
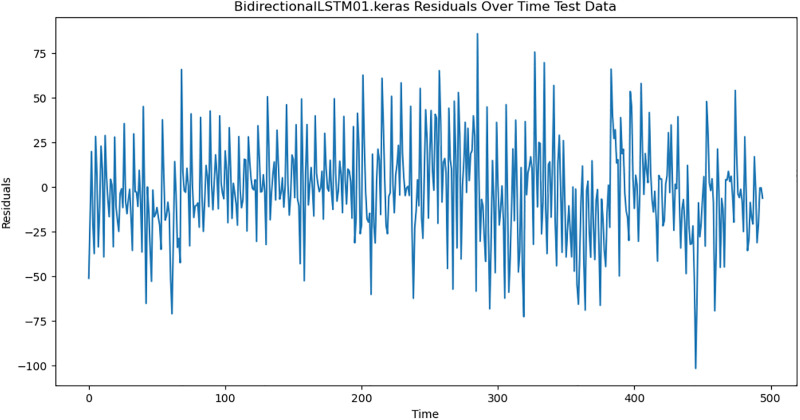
Residuals Over Time.

**Fig 51:**
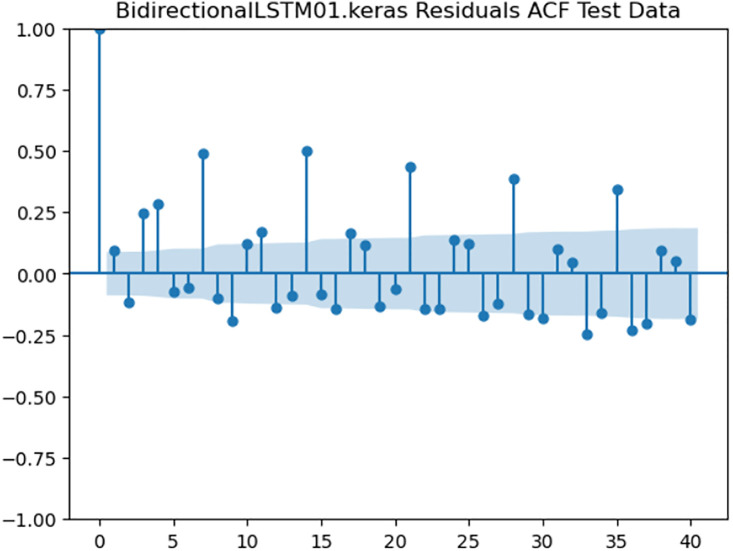
Residuals ACF Data.

**Fig 52:**
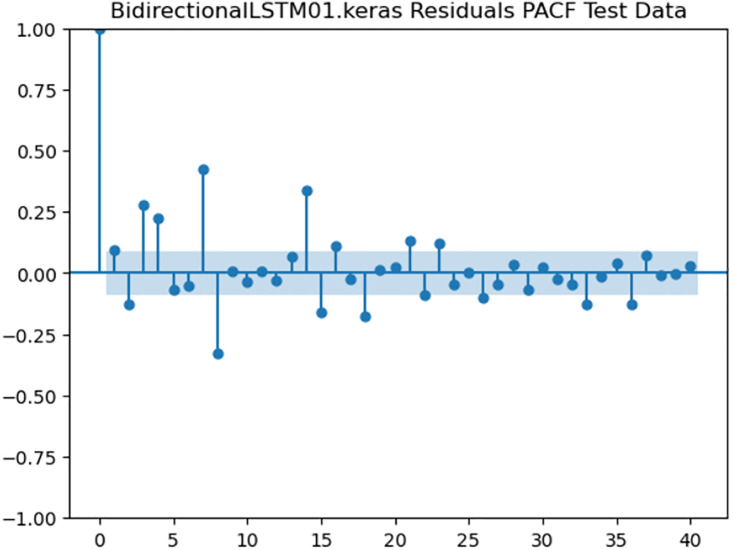
Residuals PACF Data.

**Fig 53:**
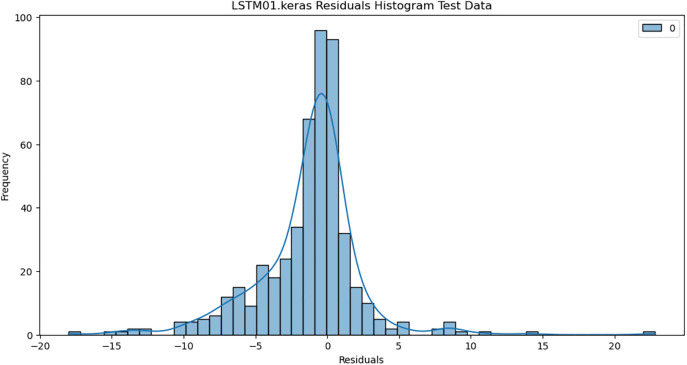
Residuals Histogram.

**Fig 54:**
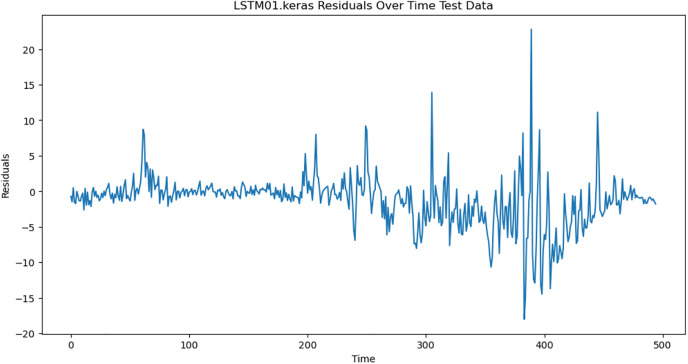
Residuals Over Time.

**Fig 55:**
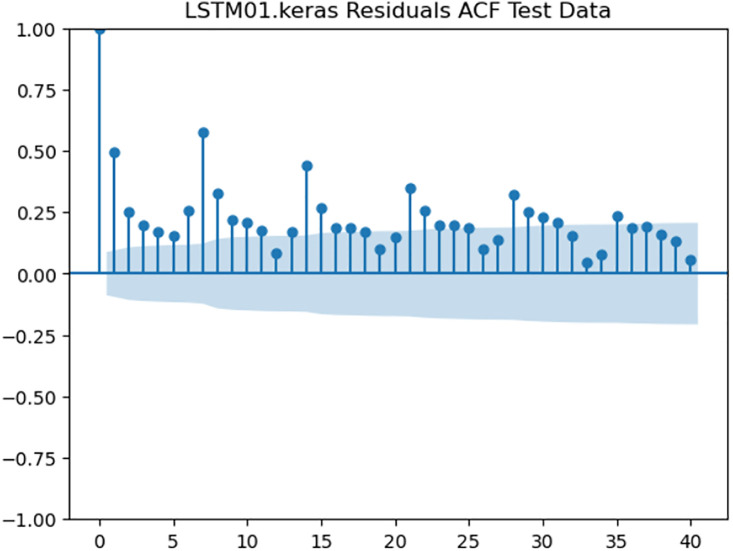
Residuals ACF Data.

**Fig 56:**
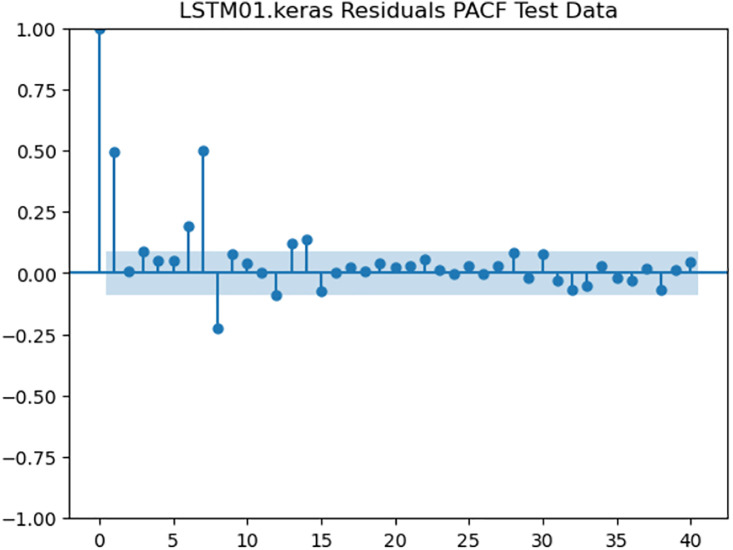
Residuals PACF Data.

#### 4.6.2. Stationarity of residuals.

Table 3 presents the results of the ADF tests for residuals from all models. The null hypothesis for the ADF test is that a time series is of unit root (i.e., it is non-stationary). If the p-value is less than a selected significance level (usually 0.05), we can reject the null hypothesis, which implies that the residuals are stationary.

From the findings, we see that the residuals of ConvLSTM, CNN-LSTM, and Bidirectional LSTM models are weakly stationary with high confidence levels (p-values < 0.05), showing that the three models manage to capture underlying data patterns efficiently without leaving residuals with strong autocorrelation. The Stacked LSTM model yields a larger p-value (0.0594), implying that its residuals are weakly stationary or on the boundary of non-stationarity. This means that the model does not necessarily reflect the temporal dependencies in the data.

#### 4.6.3. Implications and model refinements.

The stationarity of the residuals of ConvLSTM, CNN-LSTM, and Bidirectional LSTM models implies that they are modeling the temporal structure of the data well. Such models can be regarded as being more robust for forecasting purposes.

The Stacked LSTM model, though performing quite well, could potentially be improved upon. Possible areas of improvement include raising the training epochs, modifying hyperparameters like the number of layers and neurons, or including more lag features.

Possible future work includes taking the analysis further by evaluating other residual diagnostics like autocorrelation function (ACF) plots, partial autocorrelation function (PACF) plots, and normality tests to further verify the quality of residuals.

Residual analysis is an important step in assessing time series forecasting models. The ADF test outcomes reveal that most of the models yield stationary residuals, implying that they are fit for forecasting.

### 4.7. External validation

External validation in this research was done on a distinct time period not included in the training or testing set. That is, we utilized night-time light observations and electricity consumption observations between January 1, 2024, and March 31, 2024. This time period was completely left out of model training and testing to provide an objective evaluation of the model’s generalizability. The same pre-processing was done for this validation set as for the main dataset.

#### 4.7.1. Present validation results.

The performance of the model on the external validation dataset was measured in terms of the Mean Absolute Error (MAE), Root Mean Squared Error (RMSE), and R^2^ score. The results were as follows:

- Stacked LSTM: MAE = 2.41, RMSE = 3.82 MU, R^2^ = 0.90
- Bidirectional LSTM: MAE = 2.16, RMSE = 2.83 MU, R^2^ = 0.94
- CNN-LSTM: MAE = 35.98, RMSE = 46.83 MU, R^2^ = 0.76
- ConvLSTM: MAE = 8.81, RMSE = 10.70 MU, R^2^ = 0.82

These findings show that Bidirectional LSTM had the best performance, having the highest R^2^ value (0.94) and lowest RMSE (2.83 MU), making it the best model to predict electricity consumption. CNN-LSTM, however, had the poorest performance with an R^2^ of 0.76 and very high RMSE of 46.83 MU.

#### 4.7.2. Discussion of findings.

The results of external validation validate that Bidirectional LSTM performs well in generalizing, having high accuracy with low error in unseen data. CNN-LSTM and ConvLSTM, however, showed declines in performance, indicating that they could be more vulnerable to unseen temporal changes. Some explanations for performance differences are:

- Seasonal changes in electricity usage in early 2024, which could not have been completely included in the training data.
- Disparities in evening light patterns, perhaps driven by short-term occurrences like power blackouts, holidays, or economic fluctuations.
- Changes in satellite imagery, wherein environmental factors like weather conditions or phases of the moon might have affected the integrity of nightlight information.

### 4.8. Practical Implementation

#### 4.8.1. Implementation strategies.

Our forecasting frameworks can be made compatible with installed energy management systems via modular structures. For instance, RESTful APIs or batch processing pipelines may be created for the purpose of allowing data exchanges between forecasting modules and grid management platforms in seamless ways. In this way, operators can receive refreshed electricity consumption forecasts, which support real-time decision-making during energy distribution and load management.

#### 4.8.2. Data acquisition.

The models utilize night light satellite imagery, i.e., VIIRS data analysed through NASA’s Black Marble product suite. These datasets are always accessible through tools such as Google Earth Engine and NASA’s LAADS-DAAC. To make them usable for real-world application, automated scripts may be written to periodically download and pre-process fresh satellite imagery. This makes the forecasting system current and capable of near-real-time analysis.

#### 4.8.3. Computational requirements.

While training deep learning models like LSTM variants may be computationally demanding, the deployment process is light and suitable for real-time or near-real-time execution. Cloud platforms (e.g., AWS, GCP) with GPU support can be utilized to execute both training and inference processes efficiently. Moreover, using optimized libraries and frameworks further minimizes computational overhead in live operation.

#### 4.8.4. Scalability.

The suggested method is scalable by nature. The process, based on geospatial processing tools such as Google Earth Engine and QGIS, is flexible for different geographic areas. When data sizes grow or when the system is implemented in new areas, the modular architecture supports scaling efficiently. Additionally, the employment of standardized data formats (e.g., HDF5, GeoTIFF) and the versatile deep learning structure enable adaptation to larger data without major reconfiguration.

#### 4.8.5. Challenges and solutions.

- Data Latency: Consumption data and satellite imagery can be delayed. To counter this, continuous monitoring and automated ingestion pipelines can be set up, making sure that the forecasting system is operating with the latest available data.
- Processing Times: Preprocessing big geospatial datasets takes time. Utilizing parallel processing and cloud computing resources can effectively minimize processing times.
- Data Privacy: Electricity consumption data issues are solved by establishing strong data governance practices. This involves anonymizing data, compliance with privacy legislations, and encrypting channels of data transfer.

### 4.9. Comparison with existing models

#### 4.9.1. ARIMA models in electrical load forecasting and their robustness to noise.

This study demonstrates that ARIMA-based approaches suffer from several critical limitations: [37]

- Linearity Assumption: ARIMA assumes a linear connection in the data and hence would be unable to capture the non-linear behaviour found in electricity demand due to rampant urbanization and economic fluctuations.
- Limited External Variable Integration: Such models are not typically well-suited to incorporate external predictors like socio-economic indicators or high-resolution satellite data.

Compared to the existing methodology, the current approach benefits from state-of-the-art deep learning architectures—like variants of LSTMs—that are able to extract intricate, non-linear relationships and incorporate high-resolution Night-time Light (NTL) data. This incorporation provides a more nuanced view of urban activity and electricity consumption patterns, thus improving forecasting precision.

#### 4.9.2. Ensemble learning approaches – residential electricity consumption.

The paper “Advancing Ensemble Learning Techniques for Residential Building Electricity Consumption Forecasting: Insight from Explainable Artificial Intelligence” employs ensemble methods to improve forecasting performance. However, it exhibits several shortcomings: [38]

- Heavy Dependence on Extensive Feature Engineering: Ensemble models in this research need heavy manual involvement in engineering and choosing features, which can be time-consuming and error-prone.
- Insufficient Exogenous Data Integration: The model fails to use external variables such as satellite data indicators, possibly restricting its spatial and socio-economic variability capturing.

Through the implementation of deep learning methods, the suggested model provides automated feature extraction and seamless integration of NTL data. Apart from diminishing dependency on human-constructed feature engineering, this makes the model incorporate more contextual and relevant information and results in better and more responsive forecasts.

#### 4.9.3. Hybrid models for electricity consumption prediction.

The study “Accurate Prediction of Electricity Consumption Using a Hybrid Model” presents a hybrid forecasting approach. Although hybrid models represent an improvement over traditional techniques, some limitations remain: [39]

- Scalability and Spatial Considerations: Hybrid models in the research tend not to address spatial variability between regions explicitly, which is important for correctly predicting electricity demand in varied and fast-evolving urban environments.
- Complexity in Model Tuning: Combining various modelling approaches can bring extra complexity to parameter tuning, which can slow down real-time or near-real-time deployment.

The approach used herein addresses such issues by making use of high-resolution night-time satellite data to preserve spatial variations and by utilizing state-of-the-art deep learning frameworks that are easier to tune and scale. This produces a forecasting model that is adaptable to big datasets and has the ability to offer more sensitive predictions for varying regions.

## 5. Conclusion and future work

### 5.1. Conclusion

This study underscores the critical importance of accurate electricity consumption forecasting for effective energy management aligned with SDG 7 and 10. Leveraging night light satellite data as a proxy for economic activity, the research meticulously conducted short-term electricity consumption forecasts, demonstrating the significant impact of including the SOL on forecast accuracy. The state-wise analysis, utilizing the k-means algorithm to cluster regions based on annual SOL trends further highlighted the spatial dynamics inherent in electricity consumption patterns, resulting in enhanced forecast accuracy.

The analysis also revealed the effectiveness of different LSTM models — stacked LSTM, Bidirectional LSTM, CNN-LSTM, and ConvLSTM — for distinct forecasting purposes. While notable outcomes were achieved, the study encountered limitations such as the inability to incorporate additional variables like GDP and population due to data availability constraints on a daily basis. The challenge stemmed from the fact that GDP and population data are typically accessible only at monthly intervals, making their daily acquisition exceedingly arduous. Conversely, resorting to monthly electricity consumption and sum of lights would result in a reduction of available data points for training, thereby constraining the adaptability of deep learning methodologies.

An additional complicating factor lies in the fact that the sum of lights solely reflects the portion of electricity utilized for illuminating households, commercial areas, and streets during the night. It does not encompass the entirety of electricity consumption, including usage in industries and other scenarios where electricity consumption does not result in visible light emission.

Regarding the VIIRS satellite images, it is essential to note that the satellite’s overpass time occurs at 01:30 UTC every night. This timing poses a significant challenge as it results in a considerable loss in capturing peak light activity. Typically, human activities peak during the night, roughly between 6 PM to 11 PM local time, making the satellite’s overpass time a significant limitation.

Nevertheless, despite these limitations, the study successfully demonstrated the robust correlation between night lights and electric power consumption, providing valuable insights for future energy planning and management initiatives.

### 5.2. Future work

The study identifies several avenues for improvement to address the outlined limitations and enhance the quality of results in forecasting electricity consumption. One significant constraint highlighted is the limited number of data points available for analysis, which could be mitigated by implementing calibration techniques to extend temporal coverage from satellite imagery. By expanding the dataset size, it becomes feasible to apply more sophisticated forecasting models, potentially leading to improved accuracy. Additionally, the study suggests integrating additional data such as GDP, population, temperature, and holidays into future analyses to provide a more comprehensive understanding of electricity consumption patterns. Furthermore, including emerging trends like the increasing adoption of electric vehicles and decentralized energy generation through solar panels could offer valuable insights for more accurate forecasting.

Despite the identified limitations, the study underscores the significant relationship between night lights and electric power consumption, highlighting the potential of night light satellite imagery for forecasting energy needs. Through the application of machine learning techniques and spatial analysis, the incorporation of night lights data has shown to enhance the accuracy of electricity consumption forecasts, even at a state-wise level. While acknowledging these advancements, future research endeavours are encouraged to address the limitations and explore the integration of external variables and emerging trends to develop more robust forecasting models. By embracing these improvements, policymakers and energy planners can make informed decisions to support sustainable energy management practices and contribute to global energy efficiency goals.

## Supporting information

S1 File Supporting file for data processing. (DOCX)
