# Supplementary material for: Forecasting electricity consumption of India through nighttime satellite imagery
Source: PLoS One. 2025 Sep 25;20(9):e0327031. doi: 10.1371/journal.pone.0327031 (PMC12463292; doi:10.1371/journal.pone.0327031)
Supplement: S1 File — (DOCX) [file pone.0327031.s001.docx]

# **Supporting Information**

**Table 1: Values of the Mandatory quality flag in VNP46A2**

| **Value** | **Retrieval Quality** |
| --- | --- |
| 00 | High-quality |
| 01 | High-quality |
| 02 | Poor-quality |
| 255 | No retrieval |


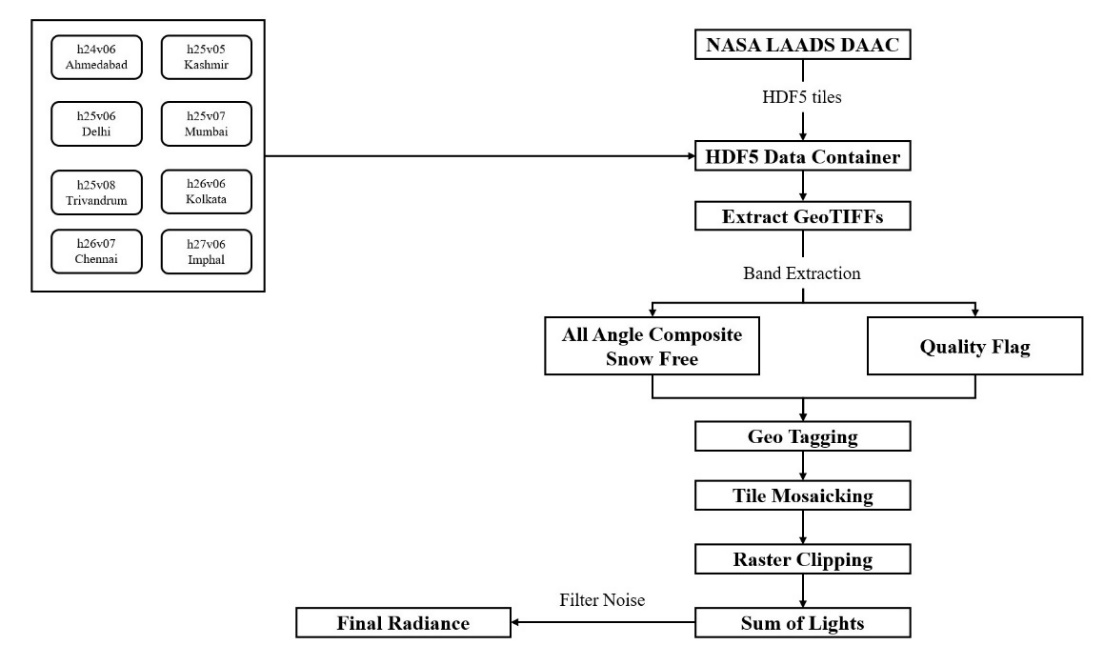


**Fig 1: Flowchart of extraction of radiance values from VIIRS satellite images**


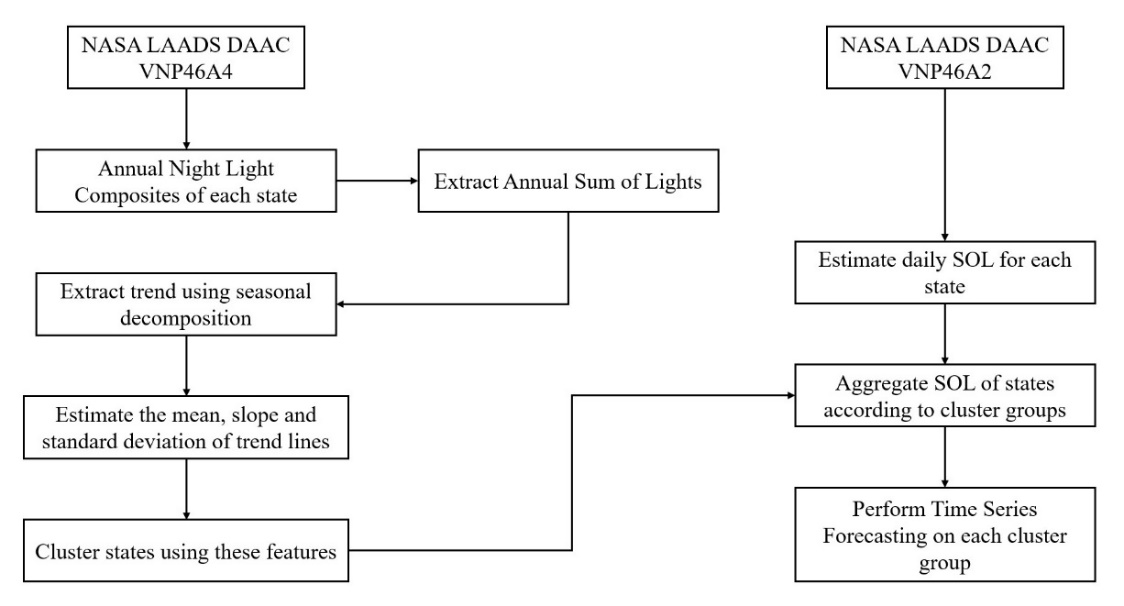


**Fig 2: Spatial analysis workflow**
